# Causes of extreme events revealed by Rényi information transfer

**DOI:** 10.1126/sciadv.adn1721

**Published:** 2024-07-26

**Authors:** Milan Paluš, Martina Chvosteková, Pouya Manshour

**Affiliations:** ^1^ Department of Complex Systems, Institute of Computer Science of the Czech Academy of Sciences, Pod Vodárenskou věží 2, 182 00 Prague 8, Czech Republic.; ^2^ Institute of Measurement Science, Slovak Academy of Sciences, Dúbravská cesta 9, 841 04 Bratislava, Slovakia.

## Abstract

Information-theoretic generalization of Granger causality principle, based on evaluation of conditional mutual information, also known as transfer entropy (CMI/TE), is redefined in the framework of Rényi entropy (RCMI/RTE). Using numerically generated data with a defined causal structure and examples of real data from the climate system, it is demonstrated that RCMI/RTE is able to identify the cause variable responsible for the occurrence of extreme values in an effect variable. In the presented example, the Siberian High was identified as the cause responsible for the increased probability of cold extremes in the winter and spring surface air temperature in Europe, while the North Atlantic Oscillation and blocking events can induce shifts of the whole temperature probability distribution.

## Teaser

The secrets behind the occurrence of extreme events are unlocked using information theory beyond Shannon.

## INTRODUCTION

Early in January 2021, very mild winter temperatures occurred in Central Europe, while Spain experienced extraordinary cold weather. For instance, on 5 January 2021, the minimum temperature in Potsdam, Germany, was 0.4°C, and in Frankfurt am Main, Germany, it was 1.7°C, while the average January temperatures there are −0.13° and 1.38°C, respectively. The average January temperatures in the Spanish cities of Madrid and Zaragoza are more than 6°C, while on 5 January 2021, the temperatures there dropped to −1.7° and −0.6°C, respectively. Three months later, many areas of France experienced devastating April night frosts. During the weeks preceding this extreme phenomenon, warm spring weather encouraged vegetation to bloom early and bud break occurred in vineyards. Then, from 5 April 2021, the temperature fell under −4°C in the early morning hours, threatening these new buds in all major wine-growing regions, e.g., Bordeaux, Champagne, or Burgundy. In Dijon, the largest city in Burgundy, the minimum daily temperatures during 7 April and 8 April 2021 were −2.8° and −4.9°C, respectively. The average April daily mean temperature there is 3°C.

Such extraordinary digressions from long-term means (“normals”) are called extreme values, or extremes, and their occurrences are called extreme events. Besides cold extremes, discussed in this study, warm extremes, heatwaves, and other meteorological and climate extreme events, such as floods, droughts, or hurricanes, have recently attracted considerable attention (*1*). Extreme events occur in diverse natural and social systems and usually have tremendous impact on human lives. Therefore, during the last decades, remarkable research effort has been devoted to understanding, modeling, and predictions of extreme events (*2*–*4*).

Researchers in any scientific discipline strive to uncover causes of observed phenomena. If studied phenomena or processes evolve in time and can be characterized by measurable quantities, registered in consecutive instants of time and stored in datasets called time series, then scientists can apply computational methods for detecting causal relations between processes represented by different datasets. Granger causality (GC thereafter) provides an approach to describe causality in quantitative, mathematically expressible terms. It was inspired by the 1950s work of the father of cybernetics, N. Wiener (*5*), and formalized by C. W. J. Granger, the 2003 Nobel Prize winner in economics (*6*). According to the GC principle, variable *C* is causal to variable *E* if the knowledge of the present (time *t*) state *C*(*t*) of the variable *C* improves the prediction of *E*(*t* + τ), i.e., of the variable *E* in a future time *t* + τ. Granger (*7*) introduced a mathematical framework for inference of causality based on linear autoregressive (AR) processes. Many approaches for causality in nonlinear processes have been proposed, based, e.g., on theory of dynamical systems (*8*–*10*), nonlinear prediction (*11*), machine learning (*12*), or data compression efficiency (*13*). One of the successful nonlinear generalizations of the GC approach is based on information theory (*14*, *15*). Mutual information *I*(*C*; *E*) measures the amount of common information contained in the variables *C* and *E*. It is computed from the probability distribution functions (PDFs) of the considered variables and can be expressed using their Shannon entropies. Possible causal influence of *C* on *E* can be evaluated using the conditional mutual information (*16*, *17*) (CMI thereafter), mathematically expressed as *I*[*C*(*t*); *E*(*t* + τ) ∣ *E*(*t*)]. (For cases of multidimensional variables, see the Supplementary Materials.) CMI or its mathematically equivalent (*15*, *17*) definition known as transfer entropy (*18*) (TE thereafter) measures the amount of information about *E*(*t* + τ) contained in *C*(*t*). The conditioning on *E*(*t*) removes the possible “present-time” common information in *C*(*t*) and *E*(*t*), to obtain the “net” information about the future of *E* contained in the presence of *C*. Studying the cause–effect relationships, various forms of CMI/TE have been successfully applied in diverse scientific disciplines (*19*), including the Earth sciences (*20*–*23*) where the approaches generalizing the GC principle are becoming increasingly popular (*24*–*30*) and successfully complementing causal counterfactual theory and data assimilation methods for the detection and attribution of weather and climate-related events (*31*, *32*).

Despite very intensive research activity in the areas of extreme events and causality, there are surprisingly few studies connecting the two topics. Zanin (*33*) proposed a metric based on conditional probability to decide whether extremes in one dataset cause extremes in another dataset. Gnecco *et al.* (*34*) connect the fields of causal inference and extreme value theory and define the causal tail coefficient (CTC) that captures asymmetries in the extremal dependence of two random variables. Dependence structures, including graphical models and directed (causal) graphs in multivariate data with extremes, were also studied (*35*–*37*).

CMI/TE is a tool from information theory (*14*), traditionally based on the Shannon entropy (*14*, *15*), which can be computed using PDF *p* of considered random variables. Rényi (*38*) proposed a more general definition of entropy that includes the term *p*^α^, i.e., the PDF taken to the power of α. The Rényi entropy is a parametric quantity in the sense that its value is influenced by the Rényi parameter α.

Jizba *et al.* (*39*) proposed to study causal information transfer between financial time series using the TE redefined in the Rényi entropy framework. They expected that certain values of the parameter α would selectively emphasize only certain sectors of the underlying PDF while strongly suppressing others. Because extreme values are typically located in so-called tails of PDF, in this study we ask whether the redefinition of CMI using the Rényi entropy concept (RCMI thereafter) could help to identify causes of extreme events. More specifically, the basic research question of this work is as follows: Considering two or more (potential) cause variables, influencing an effect variable, can the RCMI help to distinguish which of the cause variables is causing the extremes in the effect variable?

We will demonstrate that the CMI/TE redefined using the Rényi entropy framework opens the possibility to infer specifically the causes of extreme events, first using simulated data with clear cause–effect relations given by their construction. Then, we will introduce a societally relevant example of real-world data from the Earth climate system. We will consider long-term records of near-surface air temperature (SAT) from Europe as the effect variable and three potential cause variables.

The North Atlantic Oscillation (NAO) is a dominant pattern of atmospheric circulation variability in the extratropical Northern Hemisphere, and it is a major factor influencing air temperature and other meteorological variables in the Atlantic sector and surrounding continents (*40*).

Atmospheric blocking is a mid-latitude weather pattern manifesting as a quasi-stationary, long-lasting, high-pressure system that blocks or diverts the prevailing westerly large-scale atmospheric flow. Blocking events can have major impacts on the mid-latitude weather, sometimes leading to extreme events as cold spells in winter or heat waves in summer (*41*, *42*).

The Siberian High (SH) is a dominant circulation system over the Eurasian continent created by a massive collection of cold dry air that accumulates in the northeastern part of Eurasia from September until April. The SH influence, characterized by excessively low surface temperatures, affects regions extending well beyond its source area (*43*–*45*). The indices characterizing NAO, blocking events, and SH are defined in the “Climate data” section.

## RESULTS

### Simulated data

To test the ability of RCMI to uncover the known causal relations, we have numerically generated time series of three variables. There are two independent cause variables, *C*(*t*) and *X*(*t*); *C*(*t*) is a realization of an autoregressive process of order one (AR1 thereafter), and *X*(*t*) is a realization of Gaussian white noise. The effect variable *E*(*t*) is also of the AR1 type with *E*(*t*) given as a linear combination of *E*(*t* − 1) and *C*(*t* − 1). In addition, *X*(*t*) is causing extreme values in *E*(*t*) using a simple rule: If ∣*X*(*t*) ∣ > 3, then *E*(*t* + 1) = 1.8*X*(*t*)/∣*X*(*t*)∣. For details, see methods and “Simulated data” section below.

**Fig. 1.**
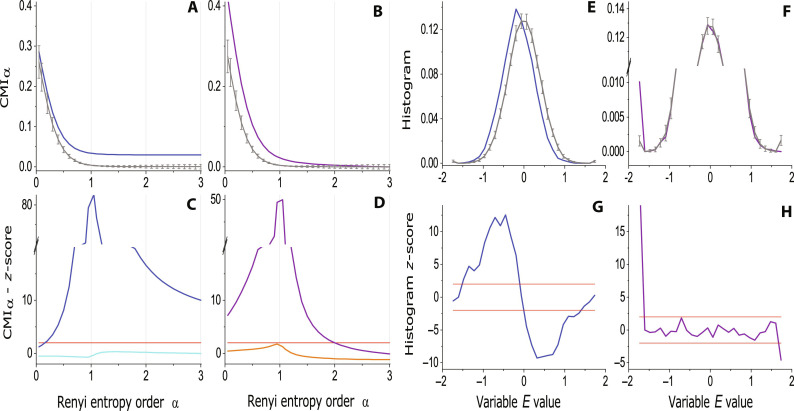
Causality in the simulated data. (**A**) The Rényi conditional mutual information (RCMI) as a function of α measuring the causality *C* → *E* (blue) and the RCMI mean ± 2 SD for the surrogate data (gray). (**B**) RCMI for the causality of extreme events *X* → *E* (purple) and the RCMI mean ± 2 SD for the surrogate data (gray). (**C**) *z*-scores for the RCMI for the causality *C* → *E* (blue) and the opposite causality *E* → *C* (turquoise). (**D**) *z*-scores for the RCMI for the causality of extreme events *X* → *E* (purple) and the opposite causality *E* → *X* (orange). The red line marks the significance level of 2 SD. (**E**) The conditional histogram of *E* given the condition *C* < −σ*_C_* (blue) and the range mean ± 2 SD of the surrogate histograms (gray). (**F**) The conditional histogram of *E* given the condition *X* < −1 (purple) and the range mean ±2 SD of the surrogate histograms (gray). (**G**) The *z*-score for the significant differences of the conditional histogram of *E* given the condition *C* < −σ*_C_* (blue). (**H**) The *z*-score for the significant differences of the conditional histogram of *E* given the condition *X* < −1 (purple). The red lines mark the significance levels of ±2 SD.

The conditional mutual information based on the Rényi entropy (RCMI), mathematically expressed as *I*_α_[*C*(*t*); *E*(*t* + 1) ∣ *E*(*t*)], quantifying the causal influence of the cause variable *C* on the effect variable *E* (in the following, the notation *C* → *E* will be used), is presented as a function of the Rényi parameter α in Fig. 1A (blue curve). The gray curve and whiskers illustrate the RCMI mean ±2 standard deviation (SD) for the surrogate data representing the null hypothesis of no causality. The difference of the RCMI of the tested data from the surrogate mean, in the number of surrogate SDs, is presented by the *z*-score in Fig. 1C using the blue curve. In the full analogy, the results for the causal relation *X* → *E* are presented in Fig. 1 (B and D), using the purple curves. For the noncausal directions, only the *z*-scores are presented (*E* → *C* in Fig. 1C, turquoise curve; and *E* → *X* in Fig. 1D, orange curve) which are confined under the red line of 2 SD, confirming no significant causality in these directions. The RCMI and the related *z*-scores in the causal directions (*C* → *E*—the blue curves in Fig. 1, A and C, respectively; and *X* → *E*—the purple curves in Fig. 1, B and D, respectively) indicate the existence of causality with a high statistical significance for large ranges of α. The *z*-scores in the causal directions in Fig. 1 (C and D) are presented with scale breaks on the ordinate to see their maxima as well as their behavior around the significance threshold line of 2 SD. On the other hand, the *z*-scores in the opposite causal directions (*E* → *C*—the turquoise curve in Fig. 1C; and *E* → *X*—the orange curve in Fig. 1D) are confined between −2 and +2 SD, confirming the unidirectionality of the detected causal relations.

Now, let us concentrate on differences between the RCMI *I*_α_ characterizing the causal influence of the two cause variables *C* and *X* on the effect variable *E*. The RCMI for *C* → *E* is statistically significant for all but the smallest values of α (Fig. 1, A and C), while the RCMI for *X* → *E* is not statistically significant for large values of α ≥ 2, but is significant for all smaller values α < 2 (Fig. 1, B and D). Because the small values of α in the expression *p*^α^ emphasize the tails of probability distributions, the statistically significant RCMI for small α is the property that we expected for the cause variables influencing the values of the effect variable on the tails of its PDF, i.e., for the cause variables causing the extreme events.

Let us illustrate the effects of the cause variables on the effect variable using the conditional probability distributions (CD thereafter), estimated as the conditional histograms. The CD of the variable *E* for the condition *C* < −σ*_C_* ($\sigma_{C}^{2}$ is the variance of *C*) is plotted in Fig. 1E (blue curve). The CD for surrogate data, representing the null hypothesis of no influence of *C* on *E*, is illustrated as mean ±2 SD in gray, and the related *z*-scores are illustrated in blue in Fig. 1G. We can see that the condition *C* < −σ*_C_* shifts the whole histogram to the left and this shift is statistically significant for all values but the values in the tails. For the variable *X*, we use the condition *X* < −1, and the results are presented in Fig. 1 (F and H). The only statistically significant effect of the variable *X* can be seen in the edge tail histogram bins; i.e., *X* influences the probability of extreme events in *E*, while *C* shifts most *E* values, but does not significantly affect the occurrence of extreme values.

The above numerical example uses Gaussian processes with artificially added extreme values. To demonstrate the performance of the introduced RCMI approach applied on data that inherently contain extreme values, we substitute the Gaussian noises by random numbers drawn from Lévy alpha-stable distributions (*46*). In the first scenario, only the variable *X* is a Lévy process, while Gaussian noise is included in the definitions of variables *C* and *E*. In a more challenging scenario, the intrinsic noise in *E* is also of the Lévy type. We demonstrate that also in these cases, the RCMI approach correctly identifies the variable *X* as the cause of extreme values in the variable *E*. Details about this extended numerical study are included in the Supplementary Materials.

### Climate data

Using simulated data from simple causality models, we have demonstrated that, indeed, using the RCMI, we can infer which cause variable is responsible for the occurrence of extreme values in the effect variable. Can we observe such a distinction in real experimental data? We will study causal relations between indices of atmospheric circulation variability modes and near-SAT *T* in Europe.

**Fig. 2.**
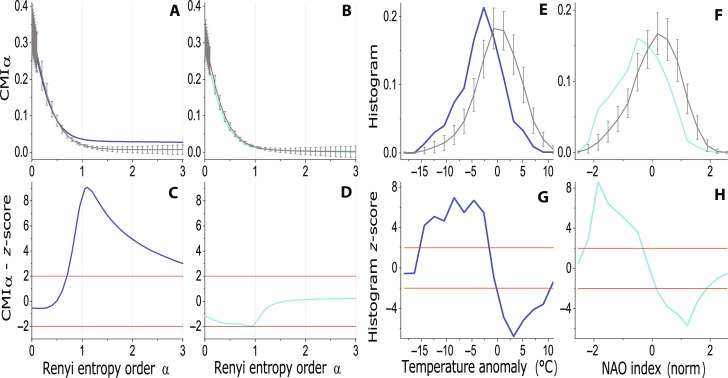
NAO and winter air temperature in Frankfurt. (**A**) RCMI as a function of α measuring the causal effect of NAO on the winter air temperature (NAO → *T*, blue) and RCMI (mean ± 2 SD) for 30 realizations of the surrogate data (gray). (**B**) RCMI for the opposite causal direction *T* → NAO (turquoise) and the related surrogate data mean ±2 SD (gray). (**C**) *z*-scores for the RCMI for the causality NAO → *T* (blue), and (**D**) *z*-scores for the RCMI for the causality *T* → NAO (turquoise). (**E**) The conditional histogram of the winter air temperature anomaly for the normalized NAO index < −1 (blue), and conditional histograms (mean ±2 SD) for 30 realizations of the surrogate data (gray), and (**G**) the related *z*-score (blue). (**F**) The conditional histogram of the winter NAO index for the normalized winter Frankfurt temperature anomaly < −1 (turquoise), and conditional histograms (mean ± 2 SD) for 30 realizations of the surrogate data (gray), and (**H**) the related *z*-score (turquoise). The red lines mark the significance levels of ±2 SD.

First, let us study the influence of NAO on the winter temperature (*T*) in Frankfurt, Germany. RCMI analysis in Fig. 2 demonstrates unidirectional causality NAO → *T* (Fig. 2, A and C; no significance in the direction *T* → NAO, Fig. 2, B and D) and RCMI is significant for NAO → *T* for α ≥ 1, while its significance disappears shortly after α decreases under 1. This observation reminds the result for the cause variable *C* in the simulation experiment above, and indeed the conditional distribution (CD) of *T* given the normalized NAO < −1 is shifted to lower values. This shift is statistically significant for the whole range of *T* values, but not for the values on the tails of the distribution (Fig. 2, E and G). Plotting the CD for the NAO index values for the condition that the normalized winter temperature anomalies in Frankfurt are smaller than −1, we can see the same effect of the distribution shift (Fig. 2, F and H). CD sees the relation of NAO and *T* as symmetrical, because CD is not a causality measure; it is just a measure of dependence. The true causality measures such as RCMI (or CMI) help us to infer that the large-scale circulation variability mode influences regional air temperature, but a single regional temperature has no measurable causal effect on the large-scale circulation variability mode, here the NAO.

**Fig. 3.**
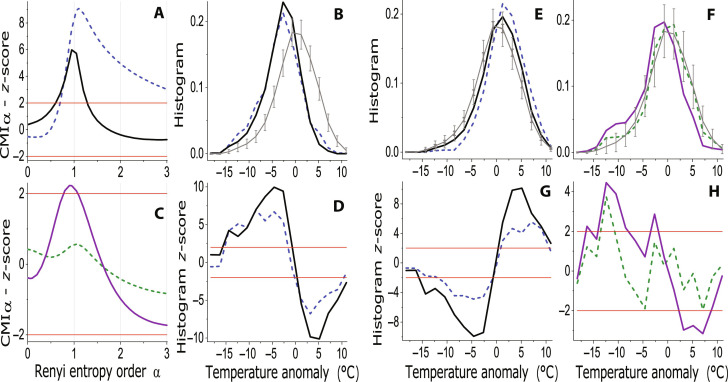
Causes of the winter air temperature in Frankfurt. (**A**) The *z*-scores for the RCMI measuring the causal effect of the blocking index on the winter air temperature (BI → *T*, black), and, for comparison, the *z*-scores for the RCMI for the causality NAO → *T* (dashed blue curve). (**B**) The conditional histogram of the winter air temperature anomaly for BI > 0 (black), and, for comparison, the conditional histogram for the normalized NAO index < −1 (dashed blue curve); and (**D**) the related *z*-scores. (**C**) The *z*-scores for the RCMI measuring the causal effect of the Siberian high on the winter air temperature (SH → *T*, dashed olive curve), and the *z*-scores for SH → *T* conditioned on the negative NAO index (SH → *T* ∣ NAO < 0, purple curve). (**E**) The conditional histogram of the winter air temperature anomaly for the normalized NAO index > 1 (dashed blue curve) and for BI = 0 (black curve), and (**G**) the related *z*-scores. (**F**) The conditional histogram of the winter air temperature anomaly for the normalized winter SH index > 1 (dashed olive curve) and for the combined condition SH index >1 and NAO < 0 (purple curve) and (**H**) the related *z*-scores. In the *z*-score graphs, the red lines mark the significance levels of ±2 SD. In the histogram graphs, the gray curves and whiskers illustrate the mean and the mean ± 2 SD for 30 realizations of the surrogate data; in (E), the thin and thick whiskers are used for the NAO and BI condition, respectively.

Let us proceed to the influence of other circulation variability indices on the Frankfurt winter temperature. The atmospheric blocking, characterized by the blocking index (BI), has a similar effect on the winter temperature as the NAO: In Fig. 3A the RCMI *z*-score for BI → *T* is plotted using the black curve, and for comparison, the dashed blue curve is used for NAO → *T*. The same color codes are used for CD in Fig. 3 (B and D), where we investigate either the BI > 0 or the NAO < −1 condition. Both the conditions induce a similar leftward shift in the temperature histogram. For completeness, in Fig. 3E, we present CD for either the BI = 0 or the NAO > 1 condition. We can see a rightward shift, smaller than the leftward shift under the previous conditions; however, the shift is still statistically significant (Fig. 3G).

There is no measurable effect of the SH itself on the Frankfurt winter temperature (SH → *T*, the dashed olive curve in Fig. 3C); however, if we consider only the days during negative NAO (NAO <0), the RCMI for SH → *T* (the purple curve in Fig. 3C) is statistically significant in a certain region under and around α = 1 (Fig. 3C). Using CD, the most apparent effect of SH is the increase of the probability of cold extremes in the range from −7° to −15°C in winter temperature anomalies (Fig. 3, F and H). In the winter temperature record from Frankfurt, as a representative of Central European stations, NAO and BI cause the shift of the whole winter temperature value distribution, while SH, under the condition of negative NAO, specifically increases the probability of the occurrence of cold extremes.

**Fig. 4.**
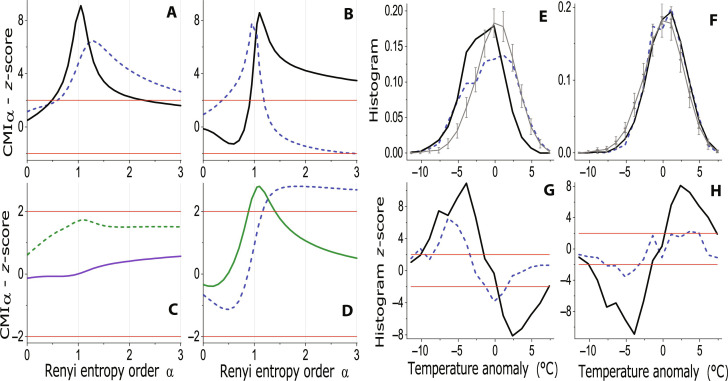
Causality in Madrid winter air temperature and circulation modes. The *z*-scores for the RCMI measuring the causal effect of (**A**) BI and NAO on the winter air temperature in Madrid (BI → *T*, black curve; NAO → *T*, dashed blue curve); (**B**) BI on NAO (BI → NAO, black curve) and NAO on BI (NAO → BI, dashed blue curve) in winter; (**C**) SH and SH during negative NAO on the winter air temperature in Madrid (SH → *T*, dashed olive curve; SH → *T* ∣ NAO < 0, purple curve); and (**D**) SH on NAO (SH → NAO, olive curve) and NAO on SH (NAO → SH, dashed blue curve) in winter. (**E**) The conditional histogram of the Madrid winter air temperature anomaly for the normalized NAO index < −1 (dashed blue curve) and BI > 0 (black curve) and (**G**) the related *z*-scores. (**F**) The conditional histogram of the Madrid winter air temperature anomaly for NAO index > 1 (dashed blue curve) and BI = 0 (black curve) and (**H**) the related *z*-scores. The red lines mark the significance levels of ±2 SD in the *z*-score graphs. The gray curves and whiskers represent the surrogate mean and mean ±2 SD range. In the combined BI/SH-conditioned histograms, the thick and thin whiskers are respectively related to the BI and SH conditions.

Let us move westward and analyze the same causes for the winter temperature in Madrid, Spain. RCMI for the causality NAO → *T* and BI → *T* (Fig. 4A) is statistically significant in similar α ranges and, although the larger significance values are not necessarily equivalent to a stronger causal effect, for the Madrid winter temperature, the main causal drive is the BI. The condition BI > 0 shifts the whole *T* probability distribution to the left; i.e., it increases the probability of cold temperature anomalies and simultaneously decreases the probability of warm anomalies (Fig. 4, E and G). On the other hand, the condition NAO < −1 increases the probability of cold anomalies but does not change the probability of warm anomalies (Fig. 4, E and G), and the positive NAO has no effect on the winter temperature in Madrid (Fig. 4, F and H). There is no causal effect of SH observed, irrespectively of the NAO condition (Fig. 4C).

We also present the relations between the studied cause variables. We can observe bidirectional causal relation between BI and NAO (Fig. 4B) and between SH and NAO (Fig. 4D). The different shapes of dependence on the parameter α stem probably from different probability distributions of the variables NAO, BI, and SH. The inference whether the observed causalities are direct or induced by some common cause is beyond the scope of this study. We just want to note that the used cause variables are not independent; however, their effects on European temperatures are different and dependent on space and time; i.e., their effects are geographically and seasonally specific. To bring more support for this statement, let us analyze causal effects in the spring temperature in Dijon, France.

**Fig. 5.**
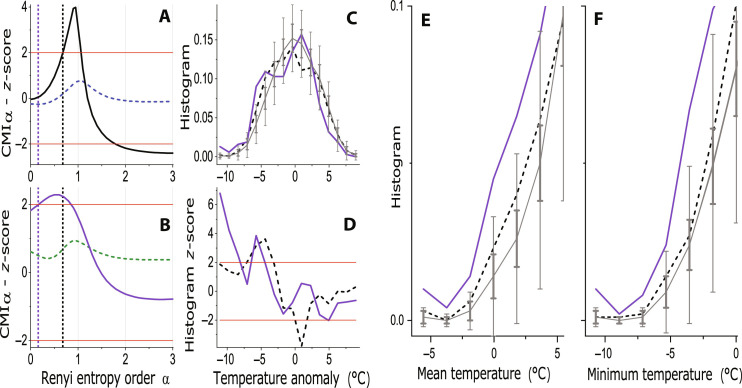
Causality in Dijon spring air temperature. The *z*-scores for the RCMI measuring the causal effect of (**A**) BI and NAO (BI → *T*, black curve; NAO → *T*, dashed blue curve) and (**B**) SH and SH during negative NAO on the spring air temperature in Dijon (SH → *T*, dashed olive curve; SH → *T* ∣ NAO < 0, purple curve). (**C**) The conditional histogram of the Dijon spring air temperature anomaly for BI > 0 (dashed black curve) and for normalized SH > 1 during negative NAO (purple curve) and (**D**) the related *z*-scores. (**E**) The left tail of the conditional histogram of the Dijon spring daily mean air temperature, and (**F**) the left tail of the conditional histogram of the Dijon spring daily minimum air temperature for BI > 0 (dashed black curve) and for normalized SH > 1 during negative NAO (purple curve). The red lines mark the significance levels of ±2 SD in the *z*-score graphs. The gray curves and whiskers represent the surrogate mean and mean ± 2 SD range. In the combined BI/SH-conditioned histograms, the thick and thin whiskers are respectively related to the BI and SH conditions.

For the Dijon spring temperature, we can see significant causality BI → *T*; however, there was no significance for the influence NAO → *T* (Fig. 5A). The influence of SH has similar behavior as in the case of the Frankfurt winter temperature—no significant causality can be detected when analyzing SH alone; however, the causality SH → *T* becomes significant for the condition of negative NAO (Fig. 5B). Note that the causality BI → *T* is significant for a narrow interval around α = 1 (Fig. 5A), while the causality SH → *T* (given NAO <0) is significant for a larger range of small α < 1 (Fig. 5B). For comparison of the ranges of small α values, for which the observed causalities are significant, we mark the left intersections of the *z*-score curves with the 2 SD significance line by vertical dashed lines in Fig. 5 (A and B). This difference of the α ranges of significant RCMI predicts the different causal effects of the two causes in the Dijon spring temperature: While BI >0 shifts leftward the center of the temperature probability distribution, i.e., the probability of small positive anomalies decreases and the probability of small negative anomalies increases; the SH > 1 condition increases the left tail of the histogram including the smallest anomalies (Fig. 5, C and D). To see this effect in usual temperature units, in Fig. 5E, we present the left tail of the same CD as in Fig. 5C, but for the spring daily mean temperatures, demonstrating that the high SH and negative NAO conditions significantly increase the probability of extreme cold daily mean temperatures under −5°C. Last, the left tail for the CD of the spring daily minimum temperature for the same condition is presented in Fig. 5F, showing the significantly increased probability of extreme spring frost around and under −10°C.

The presented results demonstrate that, indeed, the RCMI can indicate which cause variable is specifically responsible for the occurrence of extreme values. On the other hand, can we infer, just using the RCMI analysis, that some variables are not causing extremes? For the three above cause variables, NAO, BI, and SH, we will perform a simple analysis to answer the question what portion of extremes is “caused” by a particular variable. The word “caused” is given in the quotation marks, because the following computations are just a coincidence analysis, not a causality analysis.

First, we will consider cold extremes during the winter season. For an operational definition of cold extremes, we will take the first percentile of the winter air temperature distribution, i.e., 1% of the coldest winter temperature values. For instance, in the Frankfurt station air temperature data from 1950 to 2019, we have 6317 winter days; thus, we can select 63 coldest days. We ask, how many of these coldest days occurred during the NAO condition given by the normalized NAO index < −1? We find 28 days from the first percentile of the winter air temperature anomaly distribution coinciding with the NAO condition; i.e., the NAO condition “explains” or rather coincides with 44% of the cold extremes. Can such coincidence occur by chance? To find an answer, we could use random draws from the winter temperatures constrained by the number of days satisfying the NAO condition. The latter, however, occurs in clusters; therefore, we use the real NAO condition days, but we take the values from the surrogate temperature data obtained in the same way as for testing the significance in the causality analysis. We find, in 1000 surrogate data realizations, the mean 8.43 coinciding days, with the SD 4.45. The resulting *z*-score is 4.4; i.e., the original data value exceeds the surrogate mean by 4.4 SDs, and the null hypothesis of a random occurrence of the observed coincidence can be rejected.

If we use the raw air temperature data instead of the air temperature anomalies, we find 29 days from the first percentile of the raw winter air temperature distribution coinciding with the NAO condition. The related *z*-score is 4.3, rejecting the null hypothesis of a random occurrence of this coincidence. We can see that for the winter season, the results for the raw temperature and the temperature anomalies are very similar.

Continuing with the Frankfurt station winter temperature, the BI condition (BI > 0) coincides with 36 extreme cold days (*z*-score is 5.1) and the SH condition (normalized winter SH > 1) coincides with 24 extreme cold days (*z*-score is 3.4); i.e., all three studied variables “cause” (in the sense of the nonrandom coincidence) statistically significant portions of the cold winter temperature extremes.

**Fig. 6.**
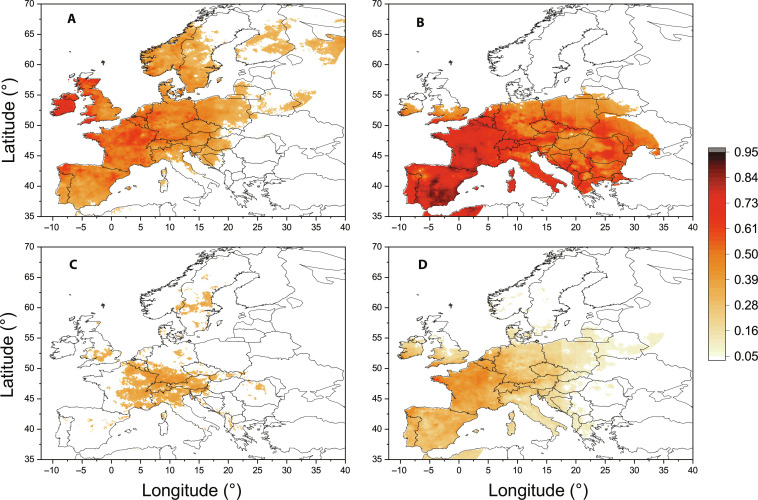
Coincidence analysis for the cold extremes in the winter air temperature in Europe. The portions of cold extremes (the first percentile of winter air temperature distribution) coinciding with (**A**) NAO condition (normalized NAO index < −1), (**B**) BI condition (BI > 0), (**C**) SH condition (normalized winter SH index > 1), and (**D**) simultaneous NAO and BI conditions. Only the statistically significant (*z*-score > 2) coincidence values are colored.

To extend this extreme coincidence analysis, we repeat the same computations using the gridded reanalysis winter temperature data and map the results in Fig. 6.

We can see that in a large part of Europe, the blocking events play a primary role in the occurrence of the cold extremes in winter temperature (Fig. 6B), while NAO is more important for the British islands and a part of Scandinavia (Fig. 6A). The SH takes its part in a smaller region of Europe, namely, in eastern France, southwestern Germany, Switzerland, Austria, Slovenia, and northern Italy (Fig. 6C). In the above analysis, we have found that the three cause variables are not independent; in particular, we have inferred a bidirectional causality between NAO and BI (Fig. 4B). On the other hand, the days of NAO and BI conditions only partially overlap. The portion of cold extremes simultaneously coinciding with both the conditions (Fig. 6D) is smaller than those coinciding with either the NAO (Fig. 6A) or the BI condition (Fig. 6B).

It should be noted that coloring of a particular grid-point in the coincidence maps was decided by the result of an individual statistical test. Because the whole maps were not corrected for multiple testing, the maps can contain also some false-positive results.

Let us move to the cold extremes in the spring season. We have observed nonnegligible differences between the results obtained using either raw or anomaly data. The reason is probably the steep increase of temperature from March to May. Thus, negative anomalies in May may still represent temperatures well above the frost point in the raw data. In the search for the causes of dangerous frosts at the beginning of the growing season, we present here the results for the raw spring temperature data.

**Fig. 7.**
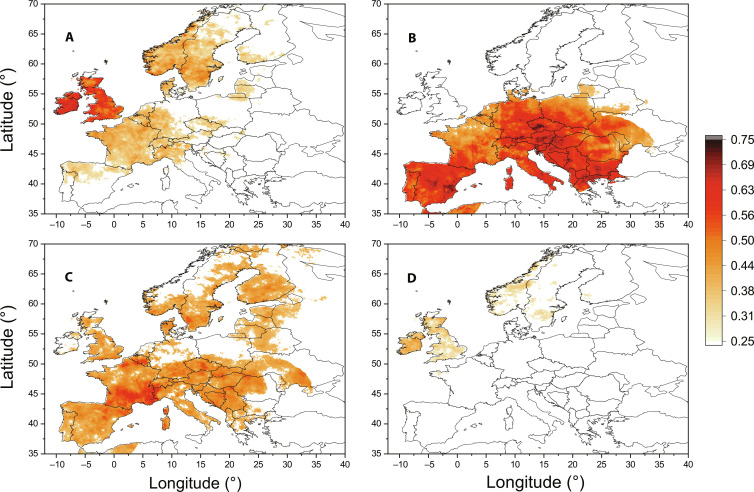
Coincidence analysis for the cold extremes in the spring air temperature in Europe. The portions of cold extremes (the first percentile of spring air temperature distribution) coinciding with (**A**) NAO condition (normalized NAO index < −1), (**B**) BI condition (BI > 0), (**C**) SH condition (normalized spring SH index > 1), and (**D**) simultaneous NAO and non-SH conditions (normalized NAO index < −1 and normalized spring SH index < 1). Only the statistically significant (*z*-score > 2) coincidence values are colored.

As the introductory example, consider the Dijon station data (1950–2019) in which we have 6440 spring days. Using again the first percentile definition of the cold extremes, we identify 64 extreme cold spring days. For the NAO condition, we find 25 coinciding values (38%, *z*-score 2.7); for the BI condition, 26 coinciding values were found (40%, *z*-score 2.5); last, the SH condition coincides with 33 cold extreme values (51%, *z*-score 2.5). In all three cases, the coincidence values are significantly higher than a random coincidence. The SH is the most important factor for the cold spring extremes in southeastern France, which can also be confirmed using the gridded reanalysis data presented in Fig. 7C. For the majority of Europe, however, the most important role is played by the blocking events (Fig. 7B), while the British islands and a part of Scandinavia are mostly influenced by the NAO. In the above causality analysis, we have found that the causal effect of the SH is detectable conditionally for nonpositive NAO (Fig. 5B). Thus, we can ask what is the “pure” effect of NAO? That is, how many cold extremes coincide with the NAO condition if the SH condition is not fulfilled, i.e., for the normalized NAO index < −1 and the normalized spring SH index < 1? In the Dijon station data, this condition coincides with 13 extreme cold values, which means 20%, and the *z*-score is 0.76. Thus, the 13 coinciding values can probably occur by chance. Applying the latter condition for the whole Europe gridded data, we observed the significant coincidence values for the pure NAO effect only in the British islands and a part of Scandinavia (Fig. 7D).

### Comparison with methods from literature

To have a comparison of the introduced RCMI approach with relevant methods from the literature, we have applied the well-known GC (*7*, *47*) approach, as well as two causal discovery methods proposed for data with extreme values, CTC (*34*) and Zanin’s causality of extreme events (*33*) (ZC), to the simulated and experimental climate data analyzed in this study.

Considering the linear AR processes *C* and *E* and Gaussian noise *X*, the GC performs well and correctly uncovers the causal relations *C* → *E* and *X* → *E*. There is even a possibility of identifying the variable causing the extreme values—after a gradual removal of extreme values from the data, the causality *C* → *E* persists, while the extreme-causing relation *X* → *E* disappears. Unfortunately, the applicability of GC is restricted to linear systems. One would expect that the linear GC would not “see” causality in nonlinear systems, i.e., would suffer by high false-negative rates; however, false-positive rates were observed. In the unidirectionally coupled Rössler systems (see the Supplementary Materials), the GC detects causality in both directions. False-positive results of this type have also been observed for other nonlinear dynamical systems by Krakovská *et al.* (*10*)

The CTC also works well for the linear AR variables *C* and *E* and Gaussian noise *X*. In the case of the nonlinear Rössler systems, CTC correctly identifies the extreme-causing relation; however, it fails to infer the correct causality between the two Rössler systems. Thus, nonlinearity in data can also be a problem for the CTC approach.

The ZC is not restricted by a linear model; however, it suffers from false-positive detections already in the case of linear AR variables *C* and *E* and Gaussian noise *X*. Although it correctly identifies the relations *C* → *E* and *X* → *E*, it also detects false causality *C* → *X*, and for some lags, also a false causality *E* → *X*. In the case of the unidirectionally coupled Rössler systems (see the Supplementary Materials), ZC indicates bidirectional causality between the two systems and an incorrect causality direction between the extreme-causing Gaussian variable and the driven Rössler system.

Next, we have applied the GC, CTC, and ZC methods to the climate data. After we have reported the results from the simulated data, the inconsistency of results from the real data is not surprising. The ZC detects bidirectional links almost everywhere, suffering apparently from high false-positive rates. The only consistent causality detection by GC and CTC is in the case of NAO influence on the winter temperature in Frankfurt. Here, GC and CTC confirm the RCMI results. In the case of NAO and Dijon spring temperature, when RCMI did not detect any causal relation, GC and CTC contradict each other. Inconsistency in detecting influence of BI and SH is probably due to nonlinearity and non-Gaussianity of these data. It should be noted, however, that CTC and ZC investigate the question “do extremes in one variable cause extremes in another variable?” That is, they require similar heavy-tailed PDFs in all studied variables, which is not the case of the studied climate data. RCMI, designed to answer the more general question “which variable is the likely cause of extremes in the affected variable?,” is not restricted by this requirement and is better suitable for real data such as those climate records studied here. Detailed presentation of these results can be found in the Supplementary Materials.

## DISCUSSION

Here, we investigated whether the RCMI is able to identify a cause variable responsible for the occurrence of extreme values in an effect variable. A simple numerical model suggested the answer “yes,” utilizing a simple causal structure of two cause variables and one effect variable, in which the effect variable *E* was linearly dependent on the cause variable *C*, and the other cause variable *X* mechanistically caused extreme values in *E*. Then, we applied the RCMI analysis on real data from the climate system: The near-SAT from European locations was considered as the effect variable, and indices of the NAO, blocking events (BI), and SH were tested as cause variables, because influences of these circulation phenomena on European air temperature have been observed (*40*–*45*). Previous time series studies, however, mostly considered the NAO and, typically, Pearson’s correlations have been computed between (mostly winter) air temperature records and NAO indices [see, e.g., (*48*)], while only a few causality analyses have been published (*13*, *49*). Causal effects of SH have been evaluated for winter SAT over northeast Asia (*50*). The analysis presented in this study has confirmed the causal influence of all three circulation phenomena on the winter and spring SAT in Europe. Moreover, we have demonstrated that RCMI can infer which cause variable is responsible for the occurrence of extreme values in the effect variable, here the SAT. The effects of NAO and BI are manifested as a shift of the whole air temperature distribution, while SH causes the increase of the probability distribution left tail, i.e., the increase of the probability of extreme cold temperatures. This conclusion, however, requires two remarks. First, the influence of SH is only observable when the NAO is not in its positive phase. Hurrell and Dickson (*51*) help us to understand this finding: “In the so-called positive phase, higher than normal surface pressures south of 55^∘^N combine with a broad region of anomalously low pressure throughout the Arctic and subarctic. Consequently, this phase of the oscillation (NAO+) is associated with stronger-than-average westerly winds across the middle latitudes of the Atlantic onto Europe….” In other words, the positive NAO brings warm Atlantic air to Europe, thus preventing the occurrence of cold extremes or even causing warm wet winter weather. The occurrence of cold extremes, especially in spring, requires the cooperation of NAO and SH, and isolated bivariate analyses of temperature T with NAO or T with SH are not sufficient. This is an example of higher-order interactions that have recently attracted attention in studies of complex systems (*52*).

The second remark stems from the above coincidence analysis. Both the NAO and BI conditions seem to explain a significant portion of cold extremes, and only in spring were we able to demonstrate that NAO has no effect without the elevated SH. It will require further research to decide whether such variables as NAO and BI are also causes of extreme events or only serve as facilitators for other causal variables. Thus, at this stage of the research, we could conclude that RCMI can provide evidence that a certain variable is the cause of extreme events, but cannot testify that other cause variables are not responsible for an increased probability of extreme event occurrence.

Here, we focused on bivariate causality analysis that cannot distinguish whether the causal effect is direct or indirect. However, for practical applications in predictions or warning systems, the bivariate causality detection is already useful. The distinction of direct causal links is necessary for understanding underlying physical mechanisms or for removal of redundant variables from predictors if the direct cause is available. In any case, the method presented here can be extended to multivariate settings and/or for including multidimensional variables (see the Supplementary Materials). Therefore the next step in this research is an implementation and testing of more effective estimation algorithms, such as those based on the k-nearest-neighbor search or kernel estimators, which have already been tested for the use in the RCMI/RTE analyses (*53*, *54*).

In the Supplementary Materials, we present detailed results of the standard GC approach, as well as two causal discovery methods proposed for data with extreme values (*33*, *34*) applied to the simulated and experimental climate data analyzed in this study. We show the capabilities and shortcomings of the methods as well as different ways of asking the research question. The RCMI method presented here does not ask whether extremes in one variable cause extremes in another variable, but which variable is the likely cause of extremes in the affected variable, regardless of the occurrence of extremes in the cause itself. Therefore, we believe that further development and applications of the RCMI open new research avenues leading to a better understanding of the occurrence of extreme events.

## MATERIALS AND METHODS

### Information-theoretic approach to causality

Consider a discrete random variable *X* with a set of values Ξ and a PDF *p*(*x*). [For simplicity, we use the notation *p*(*x*) instead of the more precise *p_X_*(*x*).] The Shannon entropy *H*(*X*) of *X* is defined as

$$H\left(X\right)=-\sum_{x\in \Xi }p\left(x\right)\;\text{log}\;p\;\left(x\right)$$ (1)

Adding another random variable *Y* with the set of values Υ, PDF *p*(*y*), and the joint PDF *p*(*x*, *y*) of both variables *X* and *Y*, we can define the joint entropy *H*(*X*, *Y*) of *X* and *Y* as

$$H\left(X,Y\right)=-\sum_{x\in \Xi }\sum_{y\in ϒ}p\left(x,y\right)\;\text{log}\;p\;\left(x,y\right)$$ (2)

By analogy, we can define the joint entropy for *n* variables.

The conditional entropy *H*(*Y*∣*X*) of *Y* given *X* is

$$H\left(Y|X\right)=-\sum_{x\in \Xi }\sum_{y\in ϒ}p\left(x,y\right)\;\text{log}\;p\;\left(y|x\right)$$ (3)

where the conditional probability *p*(*y*∣ *x*) = *p*(*x*, *y*)/*p*(*x*), for *p*(*x*) ≠ 0.

The average amount of common information, contained in the variables *X* and *Y*, is quantified by the mutual information *I*(*X*; *Y*) (*14*, *55*), defined as

I(X;Y)=H(X)+H(Y)−H(X,Y) (4)

The CMI *I*(*X*; *Y*∣*Z*) of the variables *X*, *Y* given the variable *Z* is

I(X;Y∣Z)=H(X∣Z)+H(Y∣Z)−H(X,Y∣Z) (5)

For *Z* independent of *X* and *Y*, we have

I(X;Y∣Z)=I(X;Y) (6)

By a simple manipulation, we obtain

I(X;Y∣Z)=H(X,Z)+H(Y,Z)−H(X,Y,Z)−H(Z) (7)

Equation 7 can be used to redefine the Shannonian CMI into the framework of the Rényi entropy, defined as

$$H_{\alpha }\left(X\right)=\frac{1}{1-\alpha }\text{log}\sum_{x\in \Xi }p{\left(x\right)}^{\alpha }$$ (8)

where α > 0, α ≠ 1. As α → 1, *H*_α_(*X*) converges to the Shannon entropy *H*(*X*).

We have defined CMI/RCMI for one-dimensional (scalar) variables, because this simple form has been found sufficient for uncovering unidirectional causal relations in the numerical example of variables *C*(*t*), *E*(*t*), and *X*(*t*), as well as in the experimental climate data used in this study. For the definition of higher-dimensional forms of CMI and the discussion of the need for their applications for multidimensional time series as well as Takens reconstructions (*56*) of multidimensional trajectories of dynamical systems, see the Supplementary Materials and (*17*).

### Simulated data

**Fig. 8.**
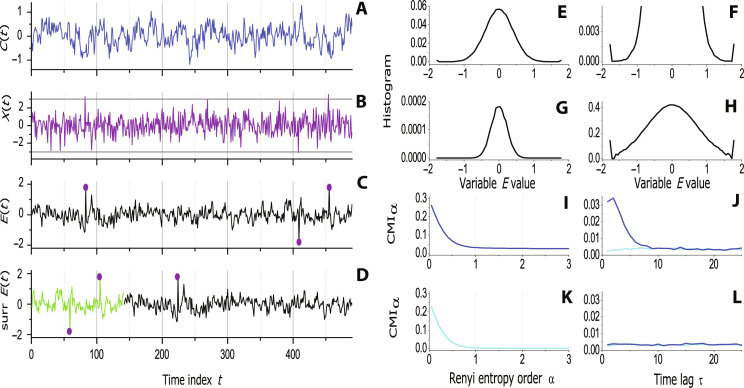
Simulated data and their characterization. (**A**) A segment of the cause variable *C*(*t*) generated by an AR1 process. (**B**) A segment of the cause variable *X*(*t*) generated as Gaussian white noise. Gray horizontal lines indicate the values ±3. (**C**) A segment of the effect variable *E*(*t*). The purple bullets mark the extreme values caused by the variable *X*(*t*) crossing the values ±3. (**D**) A realization of the circularly shifted surrogate data for the variable *E*(*t*). (**E**) The histogram (PDF) *p* of the variable *E*. (**F**) A zoomed-in view of the histogram of the variable *E*. (**G**) The third power *p*^3^ of the histogram of the variable *E*. (**H**) The 0.3th power *p*^0.3^ of the histogram of the variable *E*. (**I**) The Rényi conditional mutual information (RCMI) measuring the causal influence of the variable *C* on the variable *E* (*C* → *E*), as a function of the Rényi parameter α (blue). (**K**) RCMI in the opposite direction *E* → *C*, as a function of α (turquoise). (**J**) RCMI for α = 0.9, as a function of time lag τ, measuring the causal influence *C* → *E* (blue) and RCMI in the opposite direction *E* → *C* (turquoise). (**L**) RCMI for α = 0.9, as a function of time lag τ, measuring the causal influence of the variable *C* on the circularly shifted surrogate realization of *E* (blue) and RCMI in the opposite direction (turquoise).

Our introductory example consists of three time series. The first one, representing the cause variable *C*(*t*) (*t* = 1, … is a discrete time index), is a realization of a simple autoregressive process of order one (AR1 thereafter) (Fig. 8A, the blue curve). Its present state *C*(*t*) is given by a linear combination of its value *C*(*t* − 1) one time step back and a random number taken from a normal distribution with zero mean and unit variance

$$C\left(t\right)=a_{C}C\left(t-1\right)+\sigma_{C}\xi_{C}\left(t\right)$$

where *a_C_* = 0.7 and $\sigma_{C}^{2}$ = 0.1. The second cause variable *X*(*t*)

$$X\left(t\right)=\xi_{X}\left(t\right)$$

is a realization of white Gaussian noise with zero mean and unit variance (Fig. 8B, the purple curve). The effect variable *E*(*t*) is also of the AR1 type

$$E\left(t\right)=a_{E}E\left(t-1\right)+b_{E}C\left(t-1\right)+\sigma_{E}\xi_{E}\left(t\right)$$

where *a_E_* = 0.5, *b_E_* = 0.2, and $\sigma_{E}^{2}$ = 0.1, and all noise terms ξ*_C_*, ξ*_X_*, and ξ*_E_* are independent Gaussian random variables with zero mean and unit variance. Note that the present state *E*(*t*) is given by a linear combination of noise and previous values of both variables *E* and *C*, i.e., *E*(*t* − 1) and *C*(*t* − 1). This is a simple example of “*C* Granger causing *E*.” However, the effect variable, *E*, is influenced also by the other cause variable, *X*, in the following way: Each time *X*(*t*) is greater than 3, the value of *E*(*t* + 1) is set to 1.8. In the full analogy, *X*(*t*) < −3 causes *E*(*t* + 1) = −1.8. The effect variable *E*(*t*) is illustrated in Fig. 8C by the black curve. The extreme values ±1.8 caused by *X*(*t*) are marked by the purple bullets.

The PDF *p* of the effect variable *E*, estimated as a histogram, is presented in Fig. 8E. It is in fact a normal distribution, just on its tails—in the bins containing the extreme values ±1.8, the value of *p* is much greater than it should be in the normal distribution—see the zoomed graph in Fig. 8F. Figure 8 (G and H) illustrates the effect of taking the αth power *p*^α^ of the PDF *p*: For α = 3 (Fig. 8G), the relative weight of the most probable values around the mean (which is equal to zero here) is amplified in the cost of the weight of the values further from the mean. The situation is quite opposite for α = 0.3 (Fig. 8H)—the weight of the extreme values on the tail of the PDF is relatively increased with respect to the weight of the mean. This effect is the inspiration for considering the RCMI as a measure able to distinguish the cause variable responsible for the occurrence of extreme events.

### Estimation and statistical testing

Plugging Eq. 8 into Eq. 7, we estimate the RCMI using the simplest equidistant binning algorithm (*15*) using eight bins for each variable. Thus, the computation of RCMI (Eq. 5) requires the estimation of 3-dimensional PDF discretized into 512 bins.

RCMI *I*_α_[*C*(*t*); *E*(*t* + 1) ∣ *E*(*t*)] quantifying the causal influence of the cause variable *C* on the effect variable *E* (in the used notation *C* → *E*) is presented as a function of the Rényi parameter α in Fig. 8I (blue curve). The (nonexisting) causality in the opposite direction *E* → *C*, quantified by RCMI *I*_α_[*E*(*t*); *C*(*t* + 1) ∣ *C*(*t*)], is presented in Fig. 8K (turquoise curve). Although the values of RCMI for the causal direction *C* → *E* are greater than those for the direction *E* → *C* without any causal influence, in both the cases, the RCMI values are greater than zero and the shapes of the curves *I*_α_ as functions of α are similar: Reading for α decreasing from 3 to 0, RCMI is stable, then starts to slightly increase for α ≈ 1, while for α < 0.5, RCMI is characterized by a steep increase. Apparently, this is the behavior of the estimator of RCMI, which does not reflect any “physically increasing causality strength” for the decreasing parameter α. To detect really existing causality, we need to prove, with a statistical significance, that the RCMI estimate is indeed nonzero, i.e., greater than values given by possible bias and variance of the RCMI estimator. We apply an approach of computational statistics called the surrogate data test (*57*, *58*). The surrogate data represent a null hypothesis of no causality and allow one to compute the range of RCMI values obtained from data in which no causal relation is present. To find a way to construct surrogate data, let us evaluate, for a chosen α = 0.9, the dependence of *I*_α_[*C*(*t*); *E*(*t* + τ) ∣ *E*(*t*)] and *I*_α_[*E*(*t*); *C*(*t* + τ) ∣ *C*(*t*)] on the forward time lag τ (Fig. 8J). Here, we can see a distinctive difference, for small τ, between the RCMI in the causal direction *C* → *E* (blue curve) and in the noncausal direction *E* → *C* (turquoise curve). The causal effect *C* → *E* occurs with lag 1 sample; however, owing to a memory (autocorrelation) in the AR1 process, it is detectable for several values τ ≥ 1. However, for τ ≥ 10, both RCMI values become approximately the same; i.e., the causality is not more detectable. Therefore, for the testing purposes, we can use so-called circularly shifted surrogate data: While one variable is taken with the samples 1,2, …, *N*, where *N* is the number of samples, the other variable is taken as the samples *k*, *k* + 1, …, *N*,1,2, …, *k* − 1, where *k* is randomly selected from the interval (10, *N* − 10). See an example in Fig. 8D. The RCMI estimate, as a function of τ, for such surrogate data realization is presented in Fig. 8L, where the blue and turquoise curves for RCMI in both directions coincide in a low (no causality) level.

**Fig. 9.**
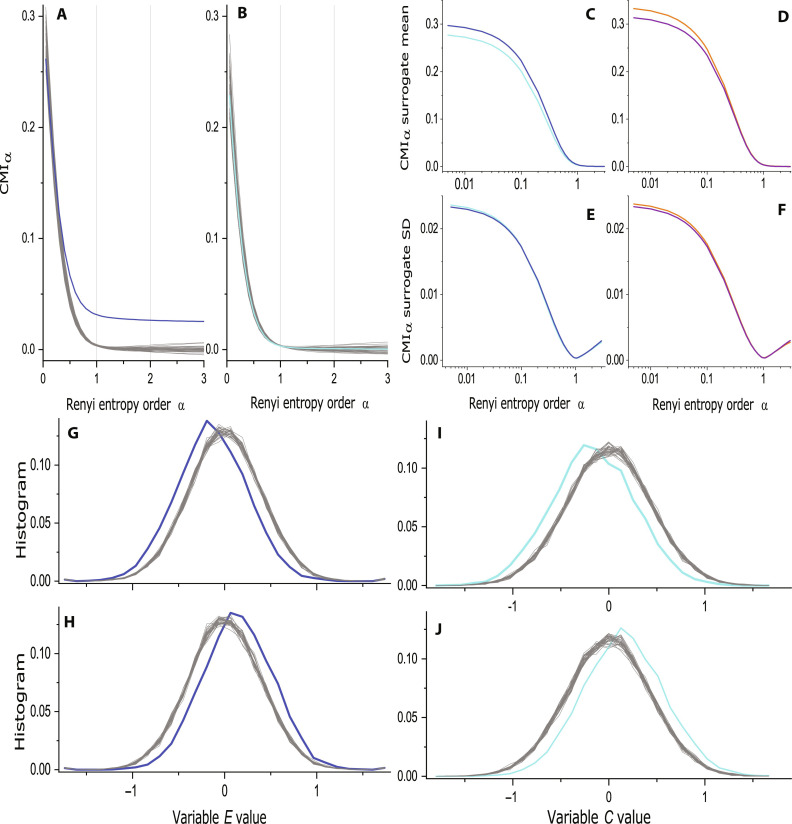
Using surrogate data. (**A**) The Rényi conditional mutual information (RCMI) as a function of α measuring the causality *C* → *E* (blue) and RCMI for 30 realizations of the surrogate data (gray). (**B**) RCMI for the opposite causality *E* → *C* (turquoise) and the related surrogate data (gray). (**C** to **F**) Statistics for RCMI of the surrogate data as functions of α in the logarithmic scale: (C) mean for the relation *C* → *E* (blue) and *E* → *C* (turquoise). (D) Mean for *X* → *E* (purple) and *E* → *X* (orange). (E) SD for *C* → *E* (blue) and *E* → *C* (turquoise). (F) SD for *X* → *E* (purple) and *E* → *X* (orange). (**G** to **J**) Conditional probability distributions (CD thereafter) estimated as histograms. CDs for 30 realizations of the surrogate data in gray. (G) CD of *E* given the condition *C* < −σ*_C_* (blue). (H) CD of *E* given the condition *C* > σ*_C_* (blue). (I) CD of *C* given the condition *E* < −σ_E_ (turquoise). (J) CD of *C* given the condition *E* > σ*_E_* (turquoise).

Let us return to the estimates of RCMI *I*_α_ plotted as a function of the parameter α. The results for the causal direction *C* → *E* are presented in Fig. 9A. The blue curve, used for the RCMI of the original *C* and *E* series, is, for a large range of α, distinctively greater than the RCMI for 30 realizations of the surrogate data, illustrated using the gray curves. On the other hand, the RCMI for the noncausal direction *E* → *C* (Fig. 9B, the turquoise curve) lies inside the bunch of the surrogate data RCMI gray curves representing the null hypothesis of no causality.

Reporting the results, we use a more practical illustration of the surrogate data range (e.g., Fig. 1A)—we do not plot the curves for individual surrogate data realizations, but using the gray curve, we present the surrogate data mean values and the gray whiskers present the range ±2 SD, where SD is the SD in 100 surrogate data realizations used in the tests here. Another useful presentation of the test results (e.g., Fig. 1C) uses the so-called *z*-score, which is defined as the difference between the (RCMI in this case) value obtained from the analyzed data and the surrogate mean, given in the number of surrogate SDs. Typically, for the *z*-score greater than 2 SD (red lines), the test result is considered statistically significant.

Let us study the behavior of the RCMI estimates from the surrogate data in detail. The increase of the RCMI estimates with the decreasing α irrespectively of the existence of causality is confirmed by the surrogate data, the means of which for all the tests are presented in Fig. 9 (C and D), focusing on small α by using the logarithmic scale on the abscissa. Note that the maxima in the four curves in Fig. 9 (C and D) differ, i.e., the positive bias in the RCMI estimates is different not only for different pairs of variables but also for different causality directions in the same pair of variables. Therefore, the good practice in causality testing, coined already by Paluš and Vejmelka (*17*), is testing each causality direction separately and avoiding the usage of differences of the RCMI (CMI and TE) estimates in the two directions as discriminating statistics. Next, Fig. 9 (E and F) presents the α dependence of the surrogate SDs. Because the surrogate SD is the denominator in the definition of the *z*-scores, the position of minima of SD explains the position of maxima of the *z*-scores in α close to 1 (e.g., Fig. 1, C and D). Thus, neither the maxima of the *z*-scores mean a “strongest causality,” but they mean that the causality tests are the most reliable for α ≈ 1.

Let us illustrate the effects of the cause variables on the effect variable using the CDs, estimated as the conditional histograms given a value of the cause variable. To quantify the difference of the conditional histogram from the distribution of *C* without any condition, we again apply the surrogate data approach. We do not use the histogram of the full dataset, but we apply the same condition on circularly shifted surrogate data in which a possible influence of the cause variable was cancelled by the way of surrogate data construction. In Results, we always summarize 100 conditional histograms computed from 100 realizations of the surrogate data and present the results as mean±2 SD using the gray curves and whiskers, as well as by plotting the related *z*-scores. However, for illustration, we plot here CDs for 30 realizations of surrogate data using the gray curves.

The conditional histogram of the variable *E* for the condition *C* < −σ*_C_* ($\sigma_{C}^{2}$ is the variance of *C*) is plotted in Fig. 9G (blue curve). We can see that the condition *C* < −σ*_C_* shifts the whole histogram of *E* to the left. In other words, keeping the values of *C* distinctly negative increases the probability of negative values of *E* and decreases the probability of positive values of *E*. Setting the condition *C* > σ*_C_* shifts the whole histogram of *E* to the right (Fig. 9H), i.e., toward the increased probability of positive values. This is the illustration of the causal effect of *C* on *E*. One can ask whether the estimation of CD cannot be used for inference of causality. Constructing the CD of *C* conditioned on values of *E* (Fig. 9, I and J) leads to the negative answer. According to the conditional histograms, the effects of *C* on *E* and of *E* on *C* are symmetrical, although there is no influence of *E* on *C*, as it is given by the data construction. Thus, the evaluation of CD can detect mutual dependence between variables, but not the direction of causal influence.

### Granger causality

A variable *X* is said to Granger-cause a variable *Y* if the prediction error of *Y* from a linear vector autoregressive model (VAR), including the past values of *Y* and *X* as predictors, is smaller than the prediction error of *Y* from a linear autoregressive model including only the own past of *Y* (*7*). The number of included past values, the model order, is usually determined by the Schwartz-Bayesian information criterion. The statistical significance of helpfulness of the variable *X* for predicting values of *Y* is established via an *F* test. Rejection of the null hypothesis of the *F* test on α confidence level (i.e., *P* value ≤ α) means that the coefficients corresponding to the past values *X* are statistically significantly different from zero in VAR, and it is concluded that *X* Granger-causes *Y*. The GC analysis can be performed using the MVGC Matlab toolbox (*47*).

### Causal tail coefficient

This causal discovery method (*34*) is tailored for situations when the causal mechanisms manifest themselves in extremes, i.e., in the tails of PDFs. The CTC was defined to reveal the causal relationship between heavy-tailed random variables, say *X* and *Y*. The definition of CTC reflects the idea that an extremely large value of *X* should cause an extreme value of *Y* in the case of a monotonic causal relationship. The causal structure can be detected by CTC if the relation between variables follows a linear structural causal model (SCM), without feedback mechanism (*34*). The method introduces, for heavy-tailed SCM, a CTC with positive values, denoted ϕ, and real-valued coefficients, denoted ψ. The knowledge of the CTC for *X* → *Y* and the opposite direction allows us to distinguish four scenarios of causal configurations:

1) *X* causes *Y* [if ψ_X → Y_ = 1 and ψ_*Y* → *X*_ ∈ (1/2,1)].

2) *Y* causes *X* [if ψ_*Y* → *X*_ = 1 and ψ_*X* → *Y*_ ∈ (1/2,1)].

3) There is a common cause [if ψ_*X* → *Y*_, ψ_*Y* → *X*_ ∈ (1/2,1)].

4) There is no causal link (if ψ_*X* → *Y*_ = ψ_*Y* → *X*_ = 1/2).

Similar results hold for ϕ. A value of CTC depends on the number of exceedances, denoted *k_n_*, where *n* represents the number of observations. A confidence interval for CTC is possible to obtain by bootstrapping the original dataset. The calculation of both heavy tail coefficients, ϕ and ψ, can be performed by the available R package at https://github.com/nicolagnecco/causalXtreme. Note that the algorithm was not developed for time series data, where the temporal order of cause and effect could help to estimate causal relationships among variables as it happens in the GC. Therefore, we recovered the direction of time from the lagged data of variables *X* and *Y* in the linear SCM. Consequently, instead of a common cause in the case ψ_*X*→*Y*_, ψ_*Y*→*X*_ ∈ (1/2,1), a bidirectional connection is detected by CTC analysis for lagged data.

### Zanin’s causality of extreme events

The causal relationship between variables *X* and *Y* is detected by analyzing how extreme events in one element correspond to the appearance of extreme events in a second one (*33*). In contrast to the causal discovery in heavy-tailed models, the method is optimized for detecting nonlinear causal relations. There are determined thresholds, denoted τ*_X_* and τ*_Y_*, for *X* and *Y*, respectively. The values that exceed the thresholds are labeled as extremes. Then, the probability that an extreme event in *Y* also corresponds to an extreme event in *X*, denoted *P*_1_, and the probability that an extreme event in *X* corresponds to an extreme event in *Y*, denoted *P*_2_, can be determined. The statistical significance of the causal connection from *X* to *Y* is evaluated by a *z* test with the null hypothesis *H*_0_ : *P*_1_ = *P*_2_ against the alternative *H_A_* : *P*_1_ > *P*_2_. The thresholds τ*_X_*, τ*_Y_* are set to obtain the lowest *P* value for the *z* test. By reversing the vector of *X*, *Y*, or both variables, the causal connection can be analyzed from the negative extremes. Zanin’s causality metric was proposed for a static dataset. On the other hand, it can be used to estimate the direction of the information flow between two time series by considering lagged data like it could be done for the CTC. Note that a large set of input values to reach stable statistics for detecting extreme events, approximately 3000 values, is required. A Python implementation of Zanin’s causality metric is freely available at www.mzanin.com/Causality.

### Climate data

Daily mean and minimum near-surface air temperature (SAT) data were provided by the European Climate Assessment & Dataset (ECA&D) project (*59*) and downloaded from https://www.ecad.eu/dailydata/predefinedseries.php (June 2021). For the presented examples, the data from the stations Frankfurt (longitude 8°35′54″E, latitude 50°02′47″N), Madrid-Retiro (3°40′41″W, 40°24′42″ N), and Dijon-Longvic (5°5′17″E, 47°16′4″N, from the period 1 January 1950 to 31 December 2019 were used. The temperature anomalies were obtained from the daily mean SAT by subtracting the annual cycle (averages for each day of the year for the whole period used). The NAO was represented by the daily values of its index obtained from the National Weather Service–Climate Prediction Center (available online at https://cpc.ncep.noaa.gov/products/precip/CWlink/pna/nao.shtml, download: March 2021).

The SH is defined using the area-averaged SH index, which was calculated by averaging the daily means of the sea level pressure (SLP) over the region between 40° – 65°N and 80° – 120°E. The dataset of SLP daily means was obtained (in June 2021) from the National Centers for Environmental Prediction-National Center for Atmospheric Research (NCEP-NCAR) Reanalysis 1 [(*60*); available online at https://psl.noaa.gov/data/gridded/data.ncep.reanalysis.surface.html]. The resolution of the dataset is 2.5° × 2.5° (latitude × longitude). The daily means of SLP were analyzed for the period 1 January 1950 to 31 December 2019.

The analysis of blocking events was also performed using the NCEP/NCAR Reanalysis 1 data (*60*). The dataset of the daily values of the 500-hPa geopotential height was obtained (in June 2021) for the period 1 January 1950 to 31 December 2019. We used the BI of Tibaldi and Molteni (*61*). Every longitude (144 values in the NCEP/NCAR Reanalysis 1) was analyzed separately. Three zonal bands of the following latitude ranges are relevant: 80° ± Δ, 60° ± Δ, and 40° ± Δ, where Δ = {−5°,0°,5°}. All data within these bands were considered, separately for the Northern Hemisphere and the Southern Hemisphere. The single necessary modification of the original method was done to the Δ setting due to different data resolution [Δ = {−4°,0°,4°} was considered in the original work (*61*)].

For the coincidence analysis (Figs. 6 and 7), the E-OBS gridded daily mean temperature dataset provided by the ECA&D (*62*) was analyzed. The version 24.0e with a 0.1° × 0.1° grid was used (downloaded in June 2021 from https://www.ecad.eu/download/ensembles/download.php). The data were analyzed for the period 1 January 1950 to 31 May 2021; the data for NAO, BI, and SH were updated till 31 May 2021 for this analysis, while the above period 1 January 1950 to 31 December 2019 was used for the causality analysis and the coincidence analysis using the station data.
