# Supplementary material for: Causes of extreme events revealed by Rényi information transfer
Source: Sci Adv. 2024 Jul 26;10(30):eadn1721. doi: 10.1126/sciadv.adn1721 (PMC11277395; doi:10.1126/sciadv.adn1721)
Supplement: Supplementary file 1 — Supplementary Text Figs. S1 to S30 Tables S1 to S3 [file sciadv.adn1721_sm.pdf]

Science Advances
AAAS

Supplementary Materials for

Causes of extreme events revealed by Rényi information transfer

Milan Paluš *et al.*

Corresponding author: Milan Paluš, mp@cs.cas.cz

Sci. Adv. **10**, eadn1721 (2024)
DOI: 10.1126/sciadv.adn1721

This PDF file includes:

Supplementary Text
Figs. S1 to S30
Tables S1 to S3

In this Supplementary material we generalize the numerical example from the main text using noises with Lévy alpha-stable distribution *(46)* in order to demonstrate the performance of the proposed method applied to data which inherently contain extreme values. Then we provide further details on the use of conditional mutual information to infer causality in multi-dimensional systems. Finally, we present detailed results of the application of the well-known Granger causality *(7, 47)* approach (GC thereafter), as well as two causal discovery methods proposed for data with extreme values, causal tail coefficient *(34)* (CTC), and Zanin's causality of extreme events *(33)* (ZC), to the simulated and experimental climate data analyzed in this study.

Causality in Lévy alpha-stable processes

Let us consider the cause variable $C(t)$ as in our first example

$$C(t) = a_C C(t-1) + \sigma_C \xi_C(t),$$

where $a_C = 0.7$, $\sigma_C^2 = 0.1$, and ξ_C are random numbers drawn from a normal distribution with zero mean and unit variance. The second cause variable $X(t)$ now will be:

$$X(t) = \eta_X(t)$$

where η_X are random numbers taken from a Lévy alpha-stable distribution *(46)*, with the stability parameter alpha equal to 1.8, skewness $\beta = 0$, scale $\gamma = 1$, and location parameter $\delta = 0$.

The effect variable $E_2(t)$ is defined as:

$$E_2(t) = a_E E_2(t-1) + 0.8C(t-1) + 0.8X(t-1) + \sigma_E \xi_E(t), \tag{S1}$$

where $a_E = 0.5$, $\sigma_E^2 = 0.1$, and the noise term ξ_E is again an independent Gaussian random variable with zero mean and unit variance. For illustration, let us define also

$$E_G(t) = a_E E_G(t-1) + 0.8C(t-1) + \sigma_E \xi_E(t),$$

i.e., the effect variable without the influence of the variable X. Realizations of these data are presented in Fig. S1. Analysis of the simulated data with Lévy noise in one cause variable is presented in Fig. S2. The Rényi conditional mutual information (RCMI) correctly identifies the causal influence in directions $C \to E_2$ and $X \to E_2$ (blue curves in Fig. S2a and Fig. S2d, respectively), and no significant causality in directions $E_2 \to C$ and $E_2 \to X$ (orange curves in Fig. S2a and Fig. S2d, respectively). As in our first example, the effect $C \to E_2$ is significant for all, but small values of α (Fig. S2a), suggesting that C has no influence on values on probability density function (PDF thereafter) tails, i.e., no effect on the occurrence of extreme values. If we used the same causal mechanism for X as in the first example in the main text, also the

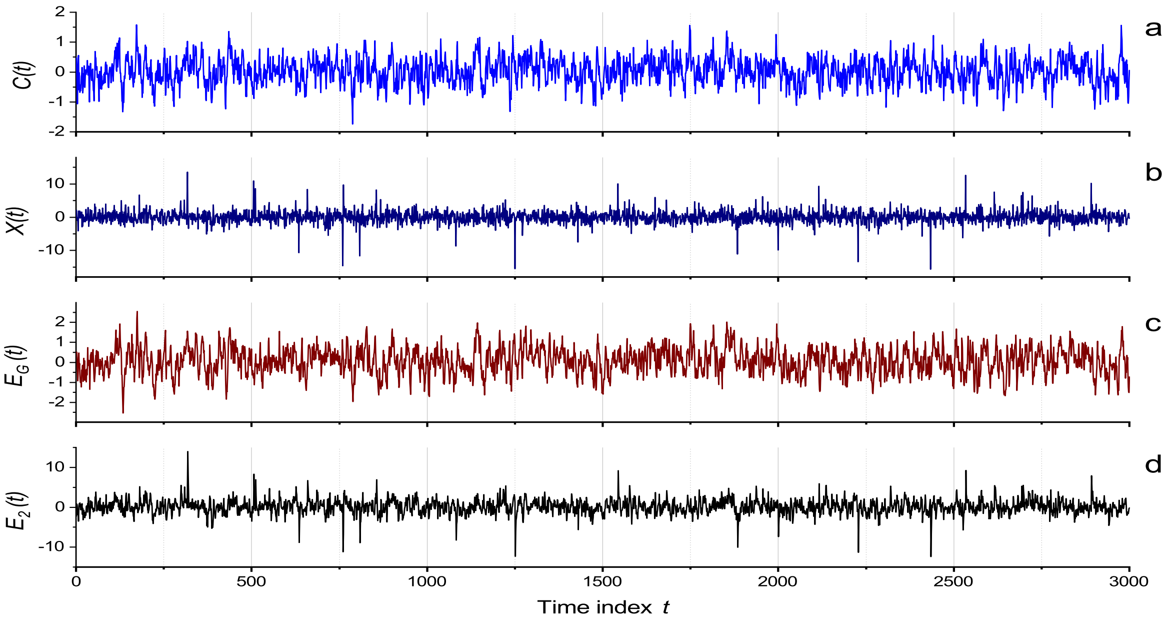

Figure S1: Simulated data with Lévy noise in one cause variable. a Independent variable C (cause), **b** cause variable X (Lévy noise), **c** effect variable E_G, driven by Gaussian noise, influenced only by C; **d** effect variable E_2, driven by Gaussian noise, influenced by both C and X.

behaviour of the RCMI Z-score was practically the same as in the first example (Fig. 1d in the main paper, not presented here). Here we present more realistic example in which the variable X enters the equation (S1) as another AR1 member. Then, the effect $X \to E_2$ is significant for all α values (Fig. S2d), i.e., X influences the whole PDF, however, it also specifically influences the PDF tails, i.e., influences the occurrence of extreme values. These findings are supported by conditional histograms. In order to better illustrate the left tails, here we present the cumulative conditional histograms. The variable C causes the PDF change just around the mean (Figs. S2b, c), while X influences both areas around the mean and also the left tail (given the condition $X < -\sigma_X$). Specifically, X increases the PDF value on its tail, i.e., X increases the probability of the occurrence of extreme values.

Let us analyze even more challenging example: Keeping the variables C and X as above, let us define

$$E_3(t) = a_E E_3(t-1) + 0.8C(t-1) + 0.8X(t-1) + \sigma_E \eta_E(t), \quad \text{(S2)}$$

where $a_E = 0.5$, $\sigma_E^2 = 0.1$, and the noise term η_E is now drawn from a Lévy alpha-stable distribution.

For illustration, let us define also

$$E_L(t) = a_E E_L(t-1) + 0.8C(t-1) + \sigma_E \eta_E(t),$$

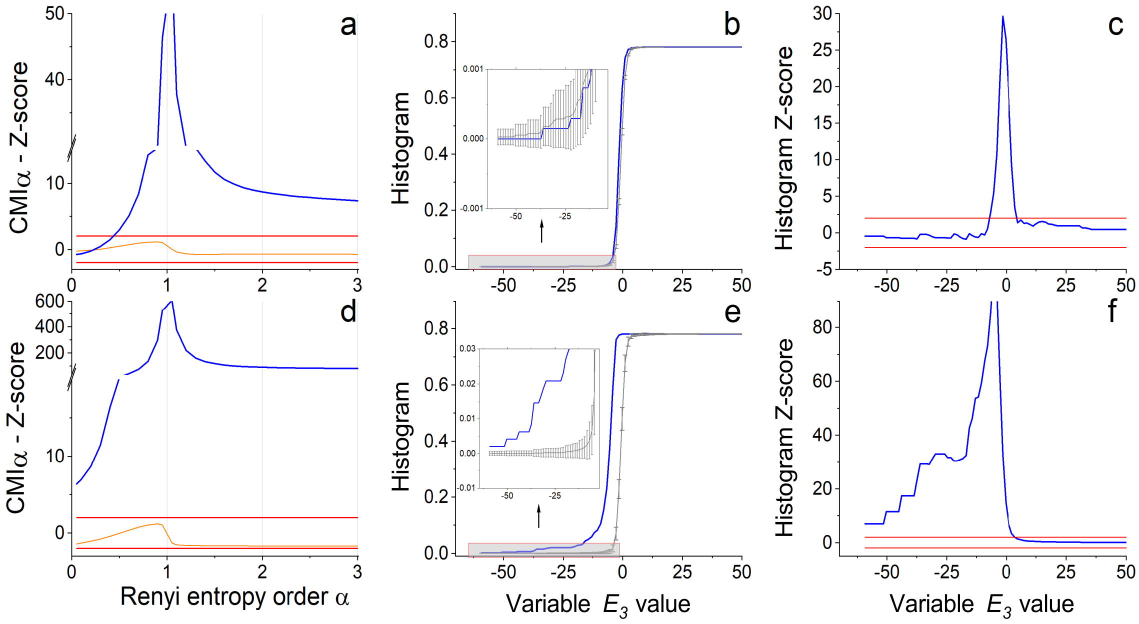

Figure S2: Causality and conditional cumulative histograms in simulated data with Lévy noise in one cause variable. **a** Z-score for the Rényi conditional mutual information (RCMI) $I_\alpha\big(C(t); E_2(t+\tau)|E_2(t)\big)$, as a function of α, measuring the causal influence of C on E_2 ($C \rightarrow E_2$, blue curve) and Z-score for the RCMI for the causality in the opposite direction $E_2 \rightarrow C$ (orange). The horizontal red lines present significance levels of $\pm$2SD. **b** Conditional cumulative histogram of E_2 given $C < -\sigma_C$, inset: zoomed view of the left tail. Blue curves illustrate results from the tested data, gray curves and whiskers present the surrogate range. **c** Z-score (blue) for the cumulative conditional histogram from **b**, red lines for $\pm$2SD of the surrogate histograms. **d** Z-score for the Rényi conditional mutual information (RCMI) $I_\alpha\big(X(t); E_2(t{+}\tau)|E_2(t)\big)$, as a function of α, measuring the causal influence of extreme causing variable X on E_2 ($X \rightarrow E_2$, blue curve) and Z-score for the RCMI for the causality in the opposite direction $E_2 \rightarrow X$ (orange). The horizontal red lines present significance levels of $\pm$2SD. **e** Conditional cumulative histogram of E_2 given $X < -\sigma_X$, inset: zoomed view of the left tail. Blue curves illustrate results from the tested data, gray curves and whiskers present the surrogate range. **f** Z-score (blue) for the cumulative conditional histogram from **e**, red lines for $\pm$2SD of the surrogate histograms.

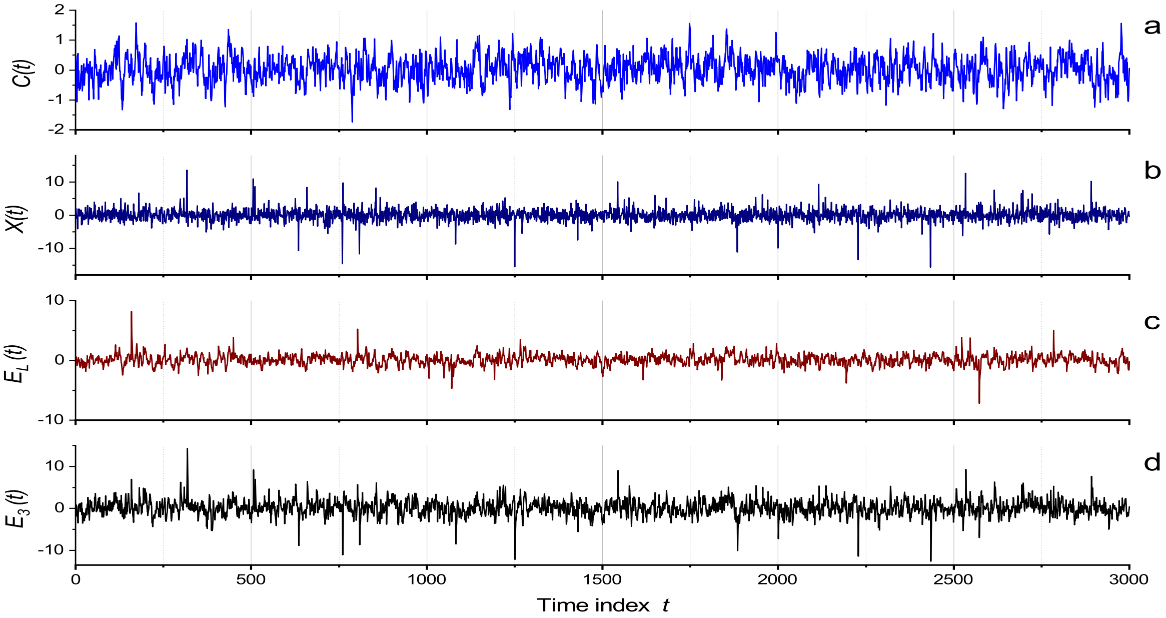

Figure S3: Simulated data with Lévy noise in one cause variable and in the effect variable. a Independent variable C (cause), **b** cause variable X (Lévy noise), **c** effect variable E_L, driven by Lévy noise, influenced only by C; **d** effect variable E_3, driven by Lévy noise, influenced by both C and X.

i.e., the effect variable without the influence of the variable X. Realizations of these data are presented in Fig. S3. Analysis of the simulated data with Lévy noise in one cause variable and in the effect variable is presented in Fig. S4.

The results of this example (Fig. S4) are practically the same as those in the previous case (Fig. S2). RCMI correctly identifies the Lévy noise variable X as the cause of extreme events in the variable E_3 even though the variable E_3 itself is driven by an independent Lévy noise and, as a consequence, E_3 contains extreme values due to its own dynamics (see variable E_L in Fig. S3). Including the influence of the variable X, the number of extreme values in E_3 increases (see variable E_3 in Fig. S3). The proposed RCMI approach correctly identifies the variable X as the variable causing the increased occurrence of extreme values in the effect variable E_3 (Fig. S4).

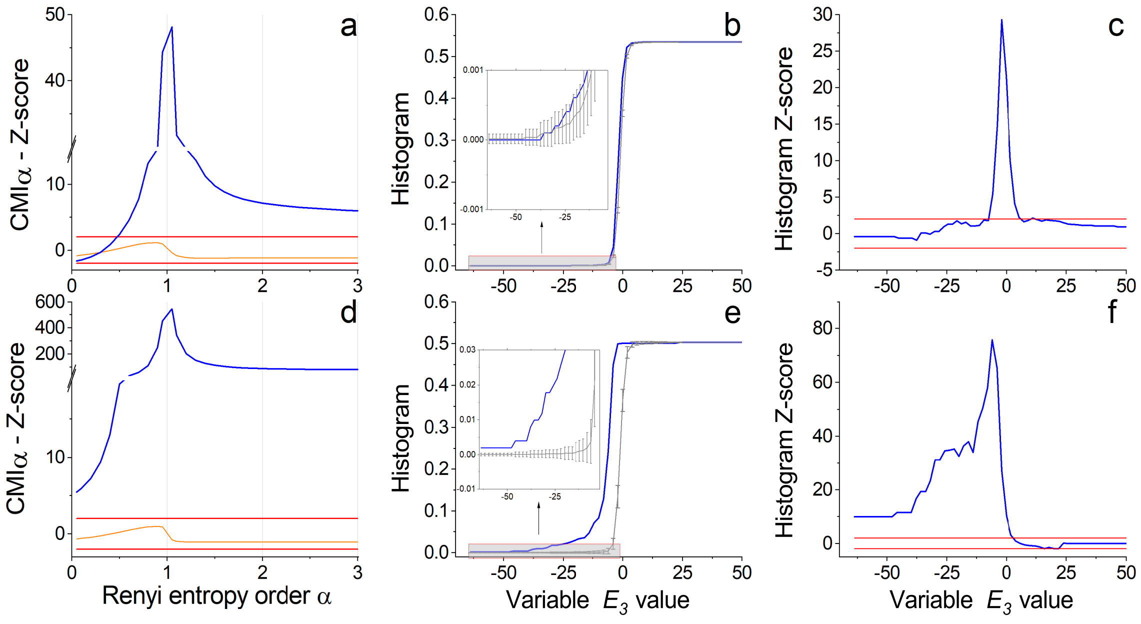

Figure S4: Causality and conditional cumulative histograms in simulated data with Lévy noise in one cause variable and in the effect variable. **a** Z-score for the Rényi conditional mutual information (RCMI) $I_\alpha\big(C(t); E_3(t+\tau)|E_3(t)\big)$, as a function of α, measuring the causal influence of C on E_3 ($C \to E_3$, blue curve) and Z-score for the RCMI for the causality in the opposite direction $E_3 \to C$ (orange). The horizontal red lines present significance levels of ±2SD. **b** Conditional cumulative histogram of E_3 given $C < -\sigma_C$, inset: zoomed view of the left tail. Blue curves illustrate results from the tested data, gray curves and whiskers present the surrogate range. **c** Z-score (blue) for the cumulative conditional histogram from **b**, red lines for ±2SD of the surrogate histograms. **d** Z-score for the Rényi conditional mutual information (RCMI) $I_\alpha\big(X(t); E_3(t+\tau)|E_3(t)\big)$, as a function of α, measuring the causal influence of extreme causing variable X on E_3 ($X \to E_3$, blue curve) and Z-score for the RCMI for the causality in the opposite direction $E_3 \to X$ (orange). The horizontal red lines present significance levels of ±2SD. **e** Conditional cumulative histogram of E_3 given $X < -\sigma_X$, inset: zoomed view of the left tail. Blue curves illustrate results from the tested data, gray curves and whiskers present the surrogate range. **f** Z-score (blue) for the cumulative conditional histogram from **e**, red lines for ±2SD of the surrogate histograms.

Conditional mutual information and inference of causality in multidimensional systems

In the Methods section of the main paper we have introduced the conditional mutual information (CMI thereafter) of three scalar variables:

$$I(X;Y|Z) = H(X,Z) + H(Y,Z) - H(X,Y,Z) - H(Z). \tag{S3}$$

Here we provide more information for better understanding of its application to the inference of causality from time series, including (projections of) trajectories of multidimensional dynamical systems.

Let $\{x(t)\}$ and $\{y(t)\}$ be time series considered as realizations of stationary and ergodic stochastic processes $\{X(t)\}$ and $\{Y(t)\}$, respectively, $t = 1, 2, 3, \ldots$. In the following we will mark $x(t)$ as x and $x(t+\tau)$ as x_τ, and the same notation holds for the series $\{y(t)\}$. The requirements of stationarity and ergodicity allow estimation of probability distributions using time averages over a single evolution of a process realization (trajectory) instead of an average over an ensemble of realizations.

The mutual information $I(y; x_\tau)$ measures the average amount of information contained in the process $\{Y\}$ about the process $\{X\}$ in its future τ time units ahead (τ-future thereafter). This measure, however, could also contain information about the τ-future of the process $\{X\}$ contained in this process itself, if the processes $\{X\}$ and $\{Y\}$ are not independent, i.e., if $I(x; y) > 0$. In order to obtain the "net" information about the τ-future of the process $\{X\}$ contained in the process $\{Y\}$ we use the conditional mutual information $I(y; x_\tau|x)$. The latter was proposed by Paluš et al. *(16)* as a possible nonlinear generalization of the Granger causality, able to detect direction of coupling of unidirectionally coupled dynamical systems. In the standard statistical language the time series $\{x(t)\}$ and $\{y(t)\}$ are considered as realizations of stochastic processes $\{X(t)\}$ and $\{Y(t)\}$, respectively. If the processes $\{X(t)\}$ and $\{Y(t)\}$ are substituted by dynamical systems evolving in measurable spaces of dimensions m and n, respectively, the variables x and y in $I(y; x_\tau|x)$ and $I(x; y_\tau|y)$ should be considered as $n-$ and $m-$dimensional vectors. In experimental practice, however, usually only one observable is recorded for each system. Therefore, instead of the original components of the vectors $\mathbf{X}(t)$ and $\mathbf{Y}(t)$, the time delay embedding vectors according to Takens *(56)* are used. Then, back in the time-series representation, we have

$$I\big(\mathbf{Y}(t); \mathbf{X}(t+\tau)|\mathbf{X}(t)\big) =$$

$$I\Big(\big(y(t), y(t-\rho), \ldots, y(t-(m-1)\rho)\big); x(t+\tau)|\big(x(t), x(t-\eta), \ldots, x(t-(n-1)\eta)\big)\Big), \tag{S4}$$

where η and ρ are time lags used for the embedding of the trajectories $\mathbf{X}(t)$ and $\mathbf{Y}(t)$, respectively. Formally, also $\mathbf{X}(t+\tau)$ should be expanded as $x(t+\tau), x(t-\eta+\tau), \ldots, x(t-(n-1)\eta+\tau)$, however, only information about one component $x(t+\tau)$ in the τ-future of the system $\{X\}$ is used for simplicity. The CMI characterizing the influence in the opposite direction

$I\big(\mathbf{X}(t);\mathbf{Y}(t+\tau)|\mathbf{Y}(t)\big)$ is defined in full analogy. Exactly the same formulation can be used for Markov processes of finite orders m and n. Based on the idea of finite-order Markov processes, Schreiber *(18)* has proposed a "transfer entropy" which is an equivalent expression *(15, 17)* for the conditional mutual information (S4).

Extensive numerical experience *(17)* suggest that the conditional mutual information in the form

$$I\big(y(t); x(t+\tau)|x(t), x(t-\eta), \ldots, x(t-(n-1)\eta)\big) \tag{S5}$$

is sufficient to infer coupling direction between the systems $\mathbf{Y}(t)$ and $\mathbf{X}(t)$. The dimensionality of the condition must contain full information about the state of the system $\mathbf{X}(t)$, while single components $y(t)$ and $x(t+\tau)$ are able to provide information about the directional coupling between the systems $\mathbf{Y}(t)$ and $\mathbf{X}(t)$. This property has been demonstrated by Paluš & Vejmelka *(17)* using the unidirectionally coupled Rössler systems given by the equations

$$\begin{aligned} \dot{x}_1 &= -\omega_1 x_2 - x_3 \\ \dot{x}_2 &= \omega_1 x_1 + a_1\, x_2 \\ \dot{x}_3 &= b_1 + x_3(x_1 - c_1) \end{aligned} \tag{S6}$$

for the autonomous system $\{X\}$, and

$$\begin{aligned} \dot{y}_1 &= -\omega_2 y_2 - y_3 + \epsilon(x_1 - y_1) \\ \dot{y}_2 &= \omega_2 y_1 + a_2\, y_2 \\ \dot{y}_3 &= b_2 + y_3(y_1 - c_2) \end{aligned} \tag{S7}$$

for the response system $\{Y\}$. The used values of parameters are $a_1 = a_2 = 0.15$, $b_1 = b_2 = 0.2$, $c_1 = c_2 = 10.0$, and frequencies $\omega_1 = 1.015$ and $\omega_2 = 0.985$, i.e., the two systems are similar, but not identical. The system $\{X\}$ is autonomous, evolving independently of the system $\{Y\}$, while $\{Y\}$ is influenced by $\{X\}$ through the diffusive coupling term $\epsilon(x_1 - y_1)$ in the right-hand side of the first component of the driven system $\{Y\}$. The parameter ϵ is referred to as the "coupling strength." Paluš & Vejmelka *(17)* generated time series as solutions of the system (S6, S7) for different coupling strengths ϵ and estimated the conditional mutual information in order to assess the existence of directional coupling or information transfer from the system $\{X\}$ to the system $\{Y\}$ and vice versa. They observed that, in the scalar case, CMI in both directions $X \to Y$ and $Y \to X$, i.e., $I\big(x_1(t); y_1(t+\tau)|y_1(t)\big)$ and $I\big(y_1(t); x_1(t+\tau)|x_1(t)\big)$, respectively, were positive and it was not possible to correctly infer the existing causal direction. The successful causal inference was achieved when the three-dimensional condition was used, then $I\big(x_1(t); y_1(t+\tau)|y_1(t), y_2(t), y_3(t)\big) > 0$, while $I\big(y_1(t); x_1(t+\tau)|x_1(t), x_2(t), x_3(t)\big) \approx 0$. Practically the same results were obtained when Takens *(56)* reconstructions instead of the original components were used, i.e., $I\big(x_1(t); y_1(t+\tau)|y_1(t), y_1(t-\eta), y_1(t-2\eta)\big) > 0$ and $I\big(y_1(t); x_1(t+\tau)|x_1(t), x_1(t-\eta), x_1(t-2\eta)\big) \approx 0$. The time delay η in the Takens *(56)* reconstruction is chosen as the first minimum of the auto-mutual information $I\big(x_1(t); x_1(t+\eta)\big)$ following the Fraser-Swinney recipe *(54)*. For the choice of the forward time lag τ there is no

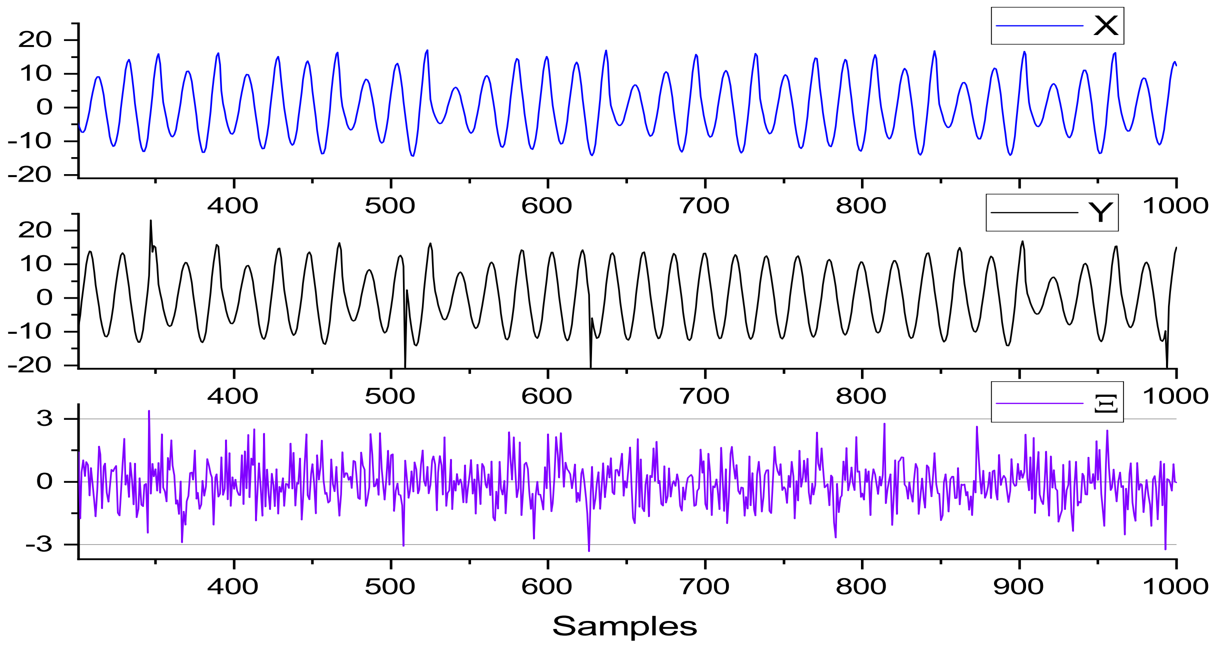

Figure S5: Simulated data from the Rössler systems with added extremes. **X** independent system (cause), **Y** influenced system (effect) with added extremes, Ξ Gaussian noise causing extremes after exceeding values ± 3.

general rule available. Therefore, it is recommended to study the dependence of CMI/RCMI on the forward time lag τ at first, and then to apply statistical testing on a selected range of time lags for which the causal influence is recognisable. It has been found *(17)*, for the systems with long-range memory and causal influence, such as the studied Rössler systems, that CMI averaged over a range of lags, e.g., $\tau = 1, \ldots, 60$, gives more robust results than those obtained using a single time lag, even if a lag which maximized CMI was chosen. For the experimental climate data, analyzed in the main paper, reasonable forward time lag τ varied from one to five days.

In this Supplementary material we chose the data from the coupled Rössler systems for a particular coupling strength $\epsilon = 0.07$. Having the original cause time series $x_1(t)$; the effect time series $y_1(t)$ was enhanced by extreme values in the same way as the variable E in the main paper. The extremes were caused by a Gausian variable, here denoted as $\Xi(t)$ using an analogous rule: if $\Xi(t) > 3$ then $y_1(t+1) = \max\{y_1(t)\} + 6$, and if $\Xi(t) < -3$ then $y_1(t+1) = \min\{y_1(t)\} - 6$. The data are illustrated in Fig. S5.

The results of the RCMI tests applied to the simulated data from the Rössler systems with added extremes are presented, in the form of the RCMI Z-scores as function of the Rényi parameter α, in Fig. S6. For the influence of Ξ on Y, the RCMI $I_\alpha\big(\xi_1(t); y_1(t+\tau)|y_1(t)\big)$ is statistically significant for all $\alpha \leq 1.7$ (Fig. S6a), while the Z-score for the RCMI in the direction $Y \to \Xi$ is confined under the significance level of 2SD (Fig. S6b). The RCMI with

the one-dimensional condition is sufficient to recognize the unidirectional causality in this case. On the other hand, the RCMI with the one-dimensional condition is not able to recognize the correct causality between the two Rössler systems: The RCMI for the both directions $X \to Y$ ($I_\alpha\big(x_1(t); y_1(t+\tau)|y_1(t)\big)$, Fig. S6c), and $Y \to X$ ($I_\alpha\big(y_1(t); x_1(t+\tau)|x_1(t)\big)$, Fig. S6d) are statistically significant for a large range of α. Then, using the three-dimensional condition, the RCMI $I_\alpha\big(y_1(t); x_1(t+\tau)|x_1(t), x_1(t-\eta), x_1(t-2\eta)\big)$ in the direction $Y \to X$ (Fig. S6f) is not significant (keeping the Z-score values between ± 2SD); and the RCMI $I_\alpha\big(x_1(t); y_1(t+\tau)|y_1(t), y_1(t-\eta), y_1(t-2\eta)\big)$ in the direction $X \to Y$ (Fig. S6e) remains statistically significant in a similar range of α as $I_\alpha\big(x_1(t); y_1(t+\tau)|y_1(t)\big)$ (Fig. S6c). Thus, the observation of Paluš & Vejmelka *(17)* that the CMI is a causality measure only for a sufficient conditioning dimension, was confirmed here also for the RCMI.

Now, let us compare the ranges of the Rényi parameter α for which the RCMI is statistically significant in the cases of two cause variable X and Ξ. Here, X is the autonomous Rössler system influencing the other Rössler system, Y, through the diffusive coupling term $\epsilon(x_1 - y_1)$. The RCMI for the causal direction $X \to Y$ (Fig. S6e) is significant for all $\alpha > 0.5$, but not significant for $\alpha < 0.5$. On the other hand, the causal effect $\Xi \to Y$ of the Gaussian variable Ξ on the system Y (Fig. S6a) is not significant for $\alpha > 1.7$, but significant for all $\alpha < 1.7$, including the smallest considered α values. Thus we can confirm the observation that the causal effect of the variable causing extreme values, i.e., increasing the probability of the tails of the probability distribution functions (PDF), is statistically significant for smaller values of the Rényi parameter α than the causal effects of variables influencing central parts of PDF.

It is important to make a technical note regarding the data requirements. The results presented in Figs. S6c–f were obtained using 8192 data samples. Paluš & Vejmelka *(17)* observed that the CMI (Shannonian framework) with the one-dimensional condition (which is applicable for the Rössler systems if the instantaneous phases *(17)* are used) yielded the 87% sensitivity for 512 samples. From about 1000 samples the CMI test reached 100% sensitivity and specificity. Here we applied the RCMI on the amplitude data which required the three-dimensional condition and, subsequently, we needed 8192 samples for the clear distinction of the unidirectional causality. On the other hand, the results for the causality between Ξ and Y in Figs. S6a,b, we used 16384 samples. When using 8192 samples, we lost the statistical significance for the causality $\Xi \to Y$ for small α (Fig. S6g). For successful detection of the causality of extremes, the data requirements are given not only by the total amount of samples, but also by the portion of extreme values contained in the data. In order to support the latter statement, we change the level for causing the extremes by the Gaussian variable Ξ from ± 3 to ± 2.8 which effectively doubles the amount of extremes in the same time series length. Now, using 8192 samples, the statistical significance for the causality $\Xi \to Y$ for small α was restored (Fig. S6i). Decreasing the amount of samples to 4096, the statistical significance for the causality $\Xi \to Y$ for small α is lost again (Fig. S6k). Therefore, in any real-data application, it is important to test the sensitivity and specificity of the RCMI causality test using suitable model data with regard to the amount of data samples as well as the portion of extreme values contained in the data. A good strategy is also using of more methods for analysis of the same data.

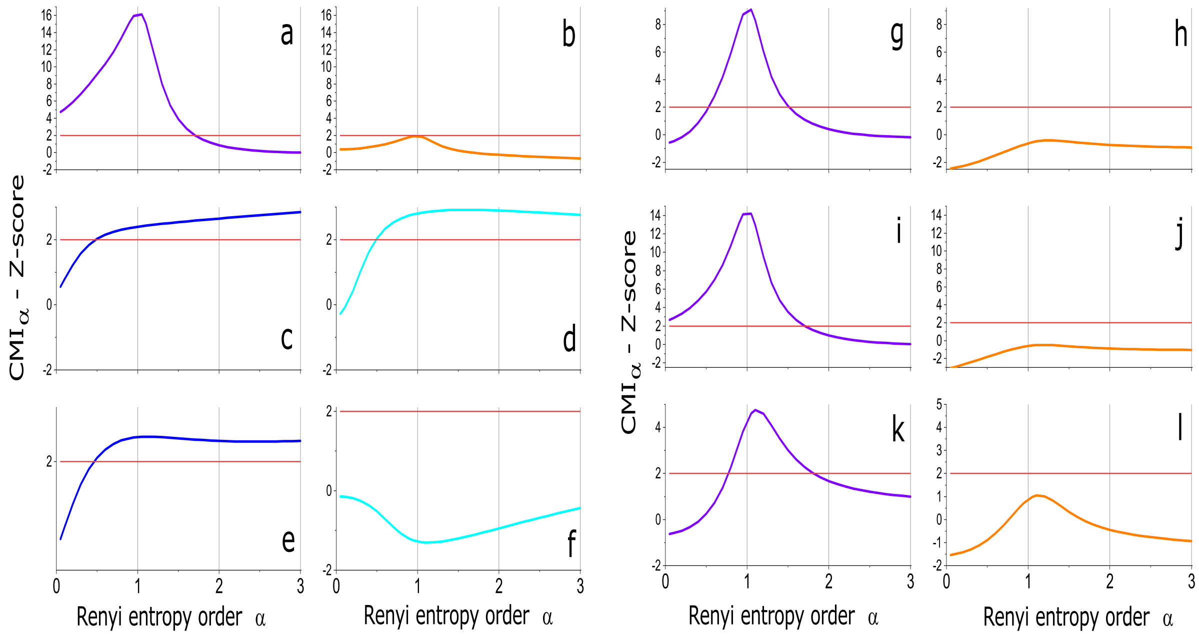

Figure S6: Causality in the simulated data from the Rössler systems with added extremes. **a** Z-score for the Rényi conditional mutual information (RCMI) $I_\alpha\big(\xi_1(t); y_1(t+\tau)|y_1(t)\big)$ as a function of α measuring the causality of extremes $\Xi \to Y$ (purple). **b** Z-score for the RCMI for the causality $Y \to \Xi$ (orange). **c** Z-score for the RCMI $I_\alpha\big(x_1(t); y_1(t+\tau)|y_1(t)\big)$ for the causality $X \to Y$ (blue) with the one-dimensional condition and **d** the opposite causality $Y \to X$, i.e. $I_\alpha\big(y_1(t); x_1(t+\tau)|x_1(t)\big)$ (turquoise). **e** Z-score for the RCMI $I_\alpha\big(x_1(t); y_1(t+\tau)|y_1(t), y_1(t-\eta), y_1(t-2\eta)\big)$ for the causality $X \to Y$ (blue) with the three-dimensional condition and **f** the opposite causality $Y \to X$, i.e. $I_\alpha\big(y_1(t); x_1(t+\tau)|x_1(t), x_1(t-\eta), x_1(t-2\eta)\big)$ (turquoise). **g, i, k** Z-score for the RCMI for the causality of extremes $\Xi \to Y$ (purple), and **h, j, l** for the opposite causality direction $Y \to \Xi$ (orange); **g, h** results for 8192 samples for the original data definition, **i, j** results for 8192 samples for the data with doubled amount of extreme values, and **k, l** results for 4096 samples for the data with doubled amount of extreme values. The red line marks the significance level of 2SD.

Results of GC, CTC and ZC methods for the simulated time series C, E, X

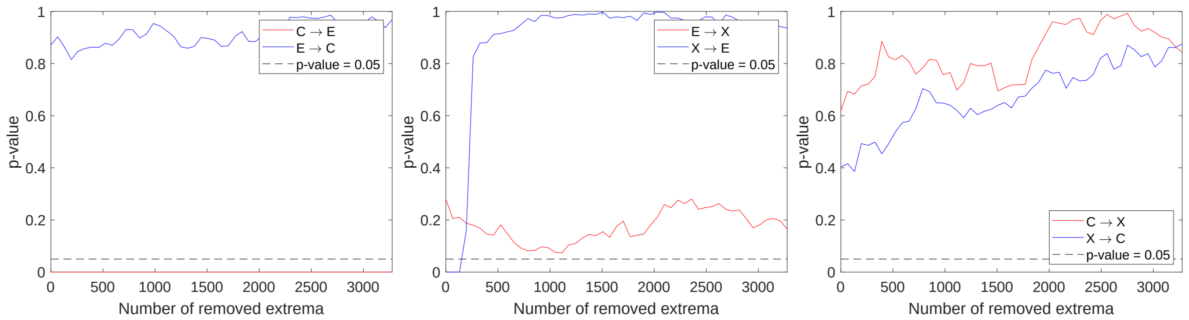

Figure S7: The results of Granger causality for the simulated series C, E, X. The detected connections $C \to E$ and $X \to E$. After removing more than 0.3% of extrema values of variable E, the connection $X \to E$ is not more presented.

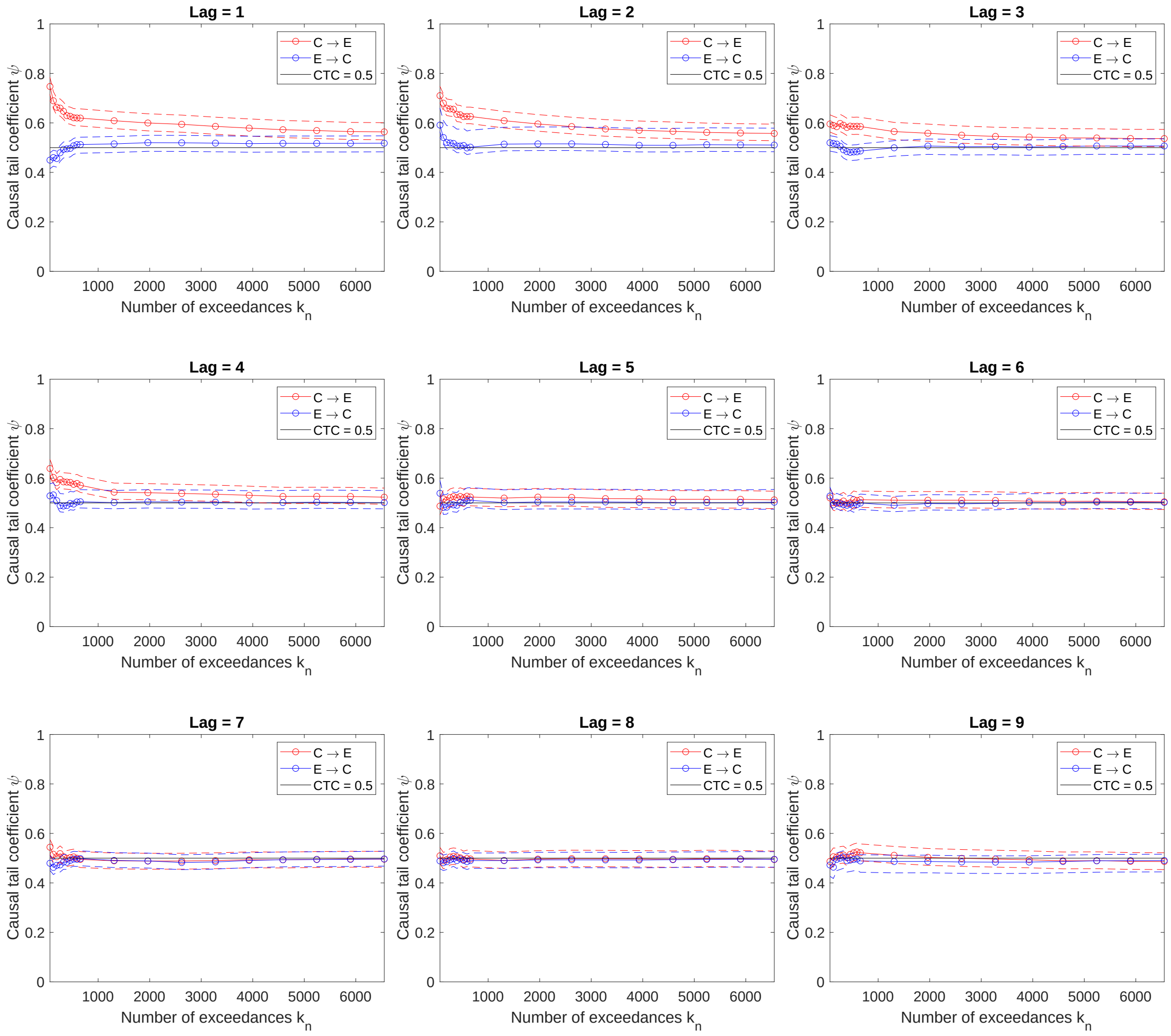

Figure S8: The estimated causal tail coefficient ψ with 95% bootstrap confidence intervals for variable C and E. Since no ψ equals to 1, there is no causality between extremes, however $\psi_{C \to E} > 0.5$ suggests causal connection $C \to E$ detected for lags 1–4.

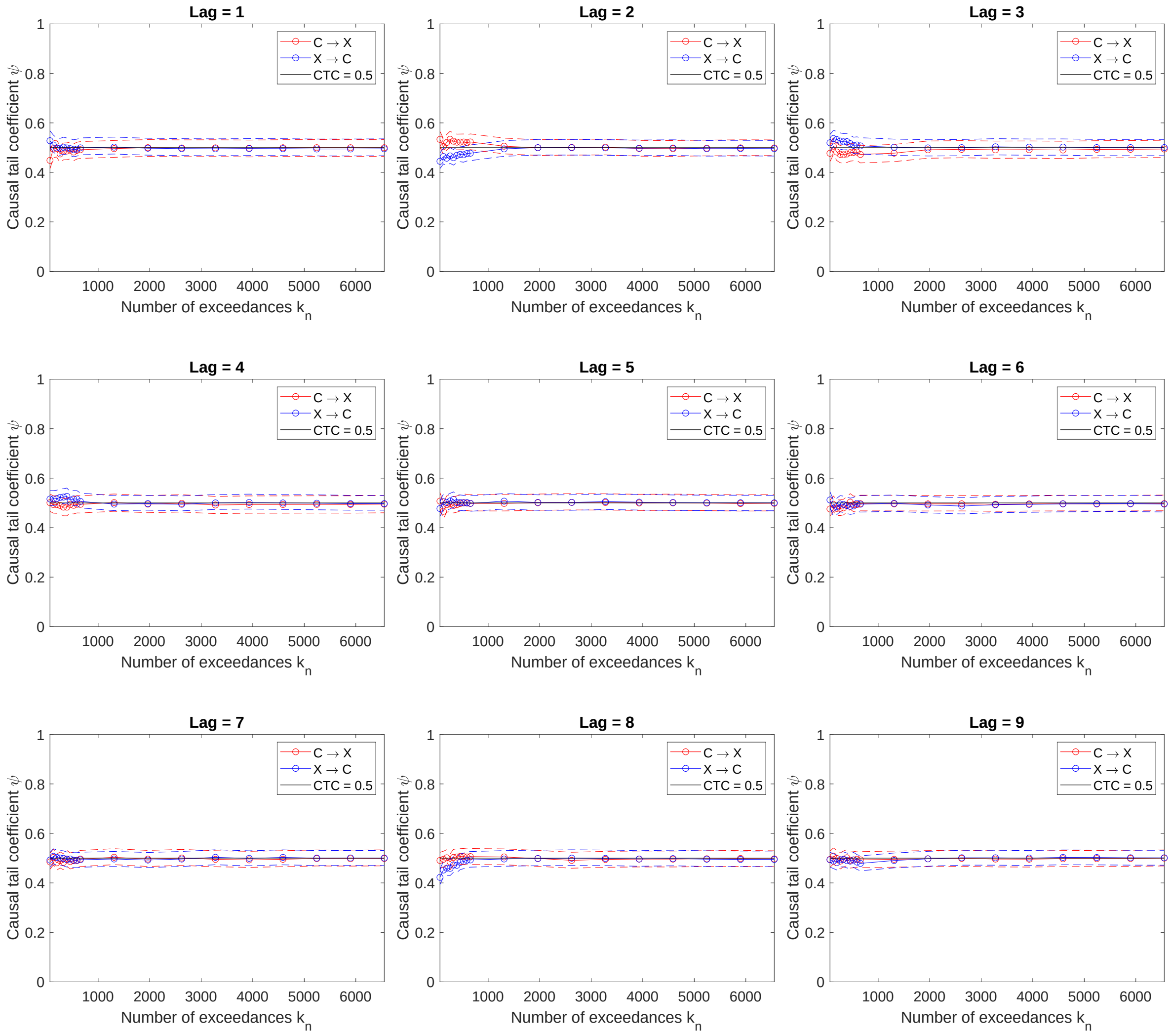

Figure S9: The estimated causal tail coefficient ψ with 95% bootstrap confidence intervals for variable C and X. The absence of a causal connection between the variables C and X was concluded.

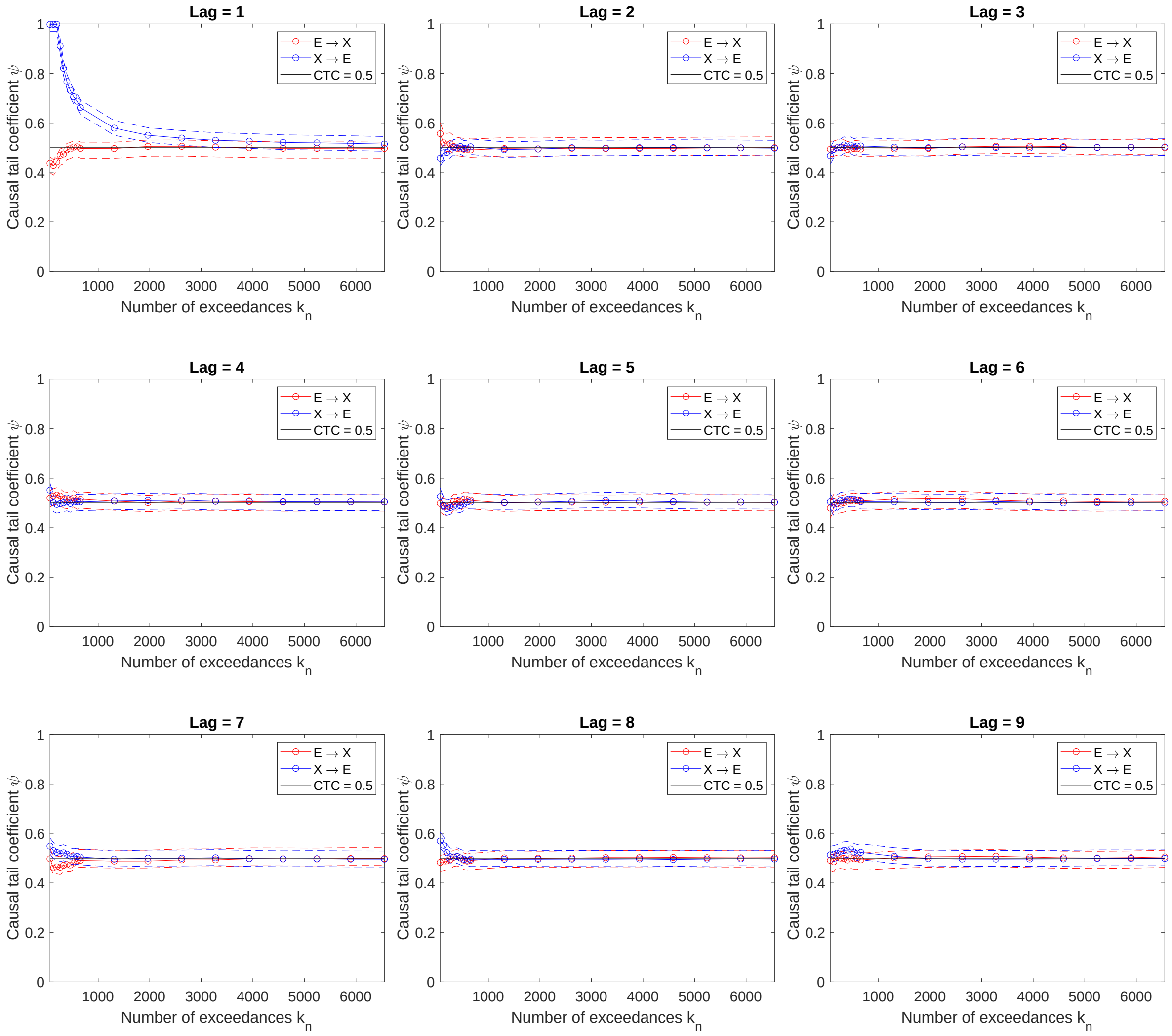

Figure S10: The estimated causal tail coefficient ψ with 95% bootstrap confidence intervals for variable E and X. The causal connection $X \to E$ detected for Lag $= 1$. The causal connection is affected by extrema, i.e., $\psi_{X \to E} = 1$.

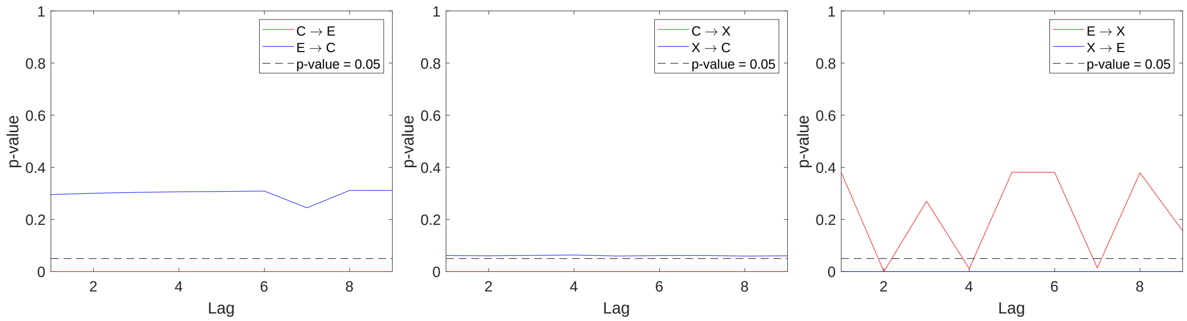

Figure S11: Results of Zanin's causality for the simulated time series C, E, X. The causal connection $C \to E$, and $C \to X$ was detected for all considered lag values. The causal connection between E and X depends on lag value.

	**GC**	**CTC**	**ZC**
C, E	$C \to E$	$C \to E$	$C \to E$
C, X	$C \perp X$	$C \perp X$	$C \to X$
E, X	$X \to E$	$X \to E$	$X \to^{*} E$

Table S1: Results of causal analysis for the simulated time series C, E, X, obtained by the Granger causality (GC), causal tail coefficients (CTC), and Zanin's causality (ZC). (*) - for some lags bidirectional connection detected.

Results of GC, CTC and ZC methods for the Rössler systems with extremes

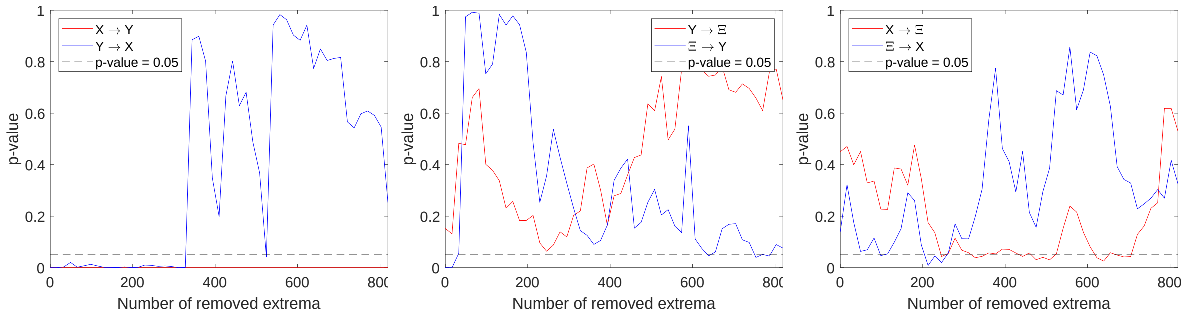

Figure S12: The results of Granger causality for the simulated time series X, Y, Ξ. The causal connections $X \leftrightarrow Y$ and $\Xi \to Y$ were detected. After removing more than 3% of extrema values of variable X, the connection $Y \to X$ is not more presented. After removing more than 0.3% of extreme values of variable Y, the connection $\Xi \to Y$ is not more presented.

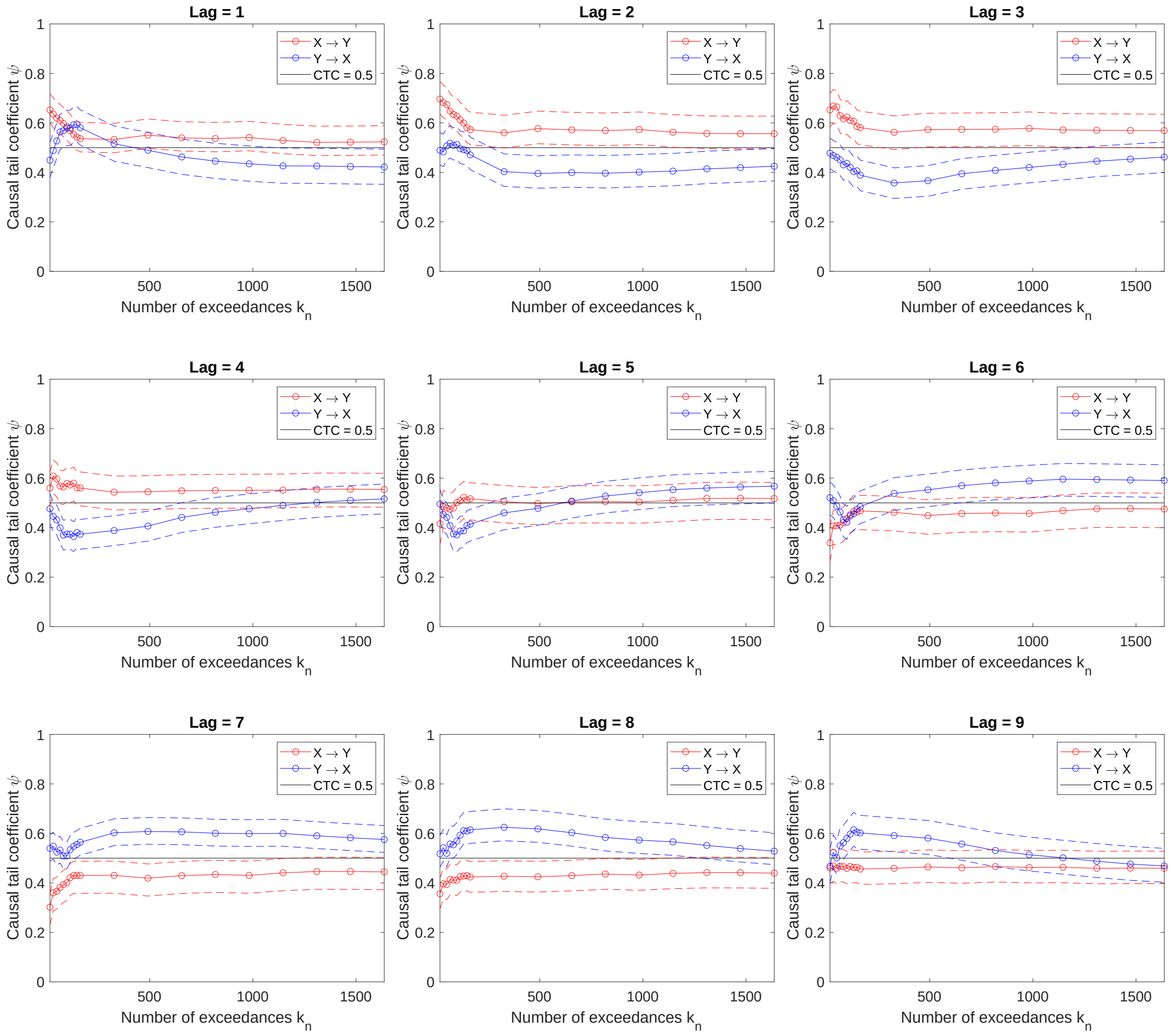

Figure S13: The estimated causal tail coefficient ψ with 95% bootstrap confidence intervals for variable X and Y. Since no ψ equals to 1, there is no causality between extremes, however $\psi_{X\to Y} > 0.5$ and $\psi_{Y\to X} = 0.5$ suggests causal connection $X \to Y$ detected for $lags$ $1-4$.

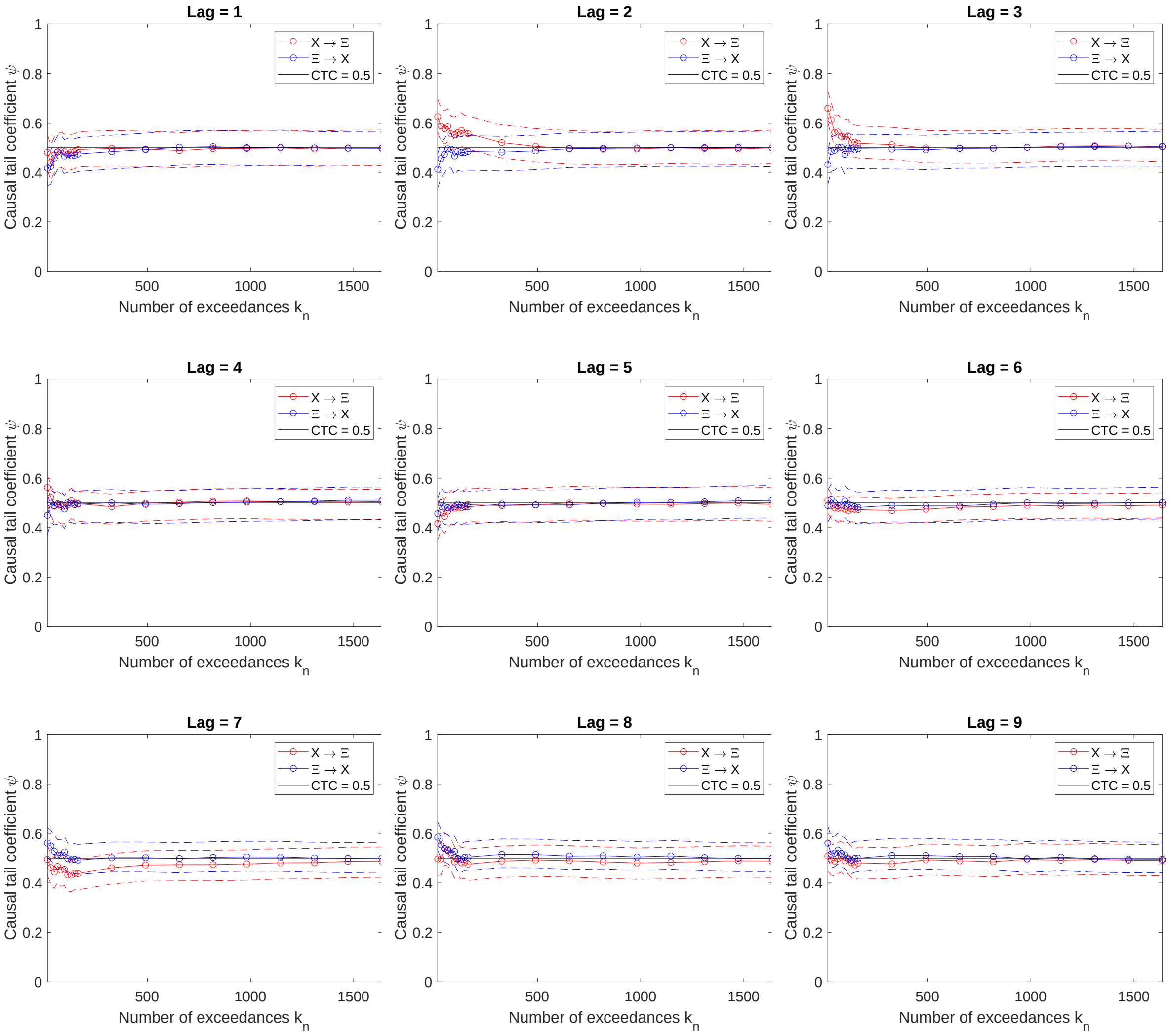

Figure S14: The estimated causal tail coefficient ψ with 95% bootstrap confidence intervals for variable X and Ξ. Since no ψ equals to 1, there is no causality between extremes, however $\psi_{X\to\Xi} > 0.5$ and $\psi_{\Xi\to X} = 0.5$ suggests causal connection $X \to \Xi$ detected for $lags$ $2-3$.

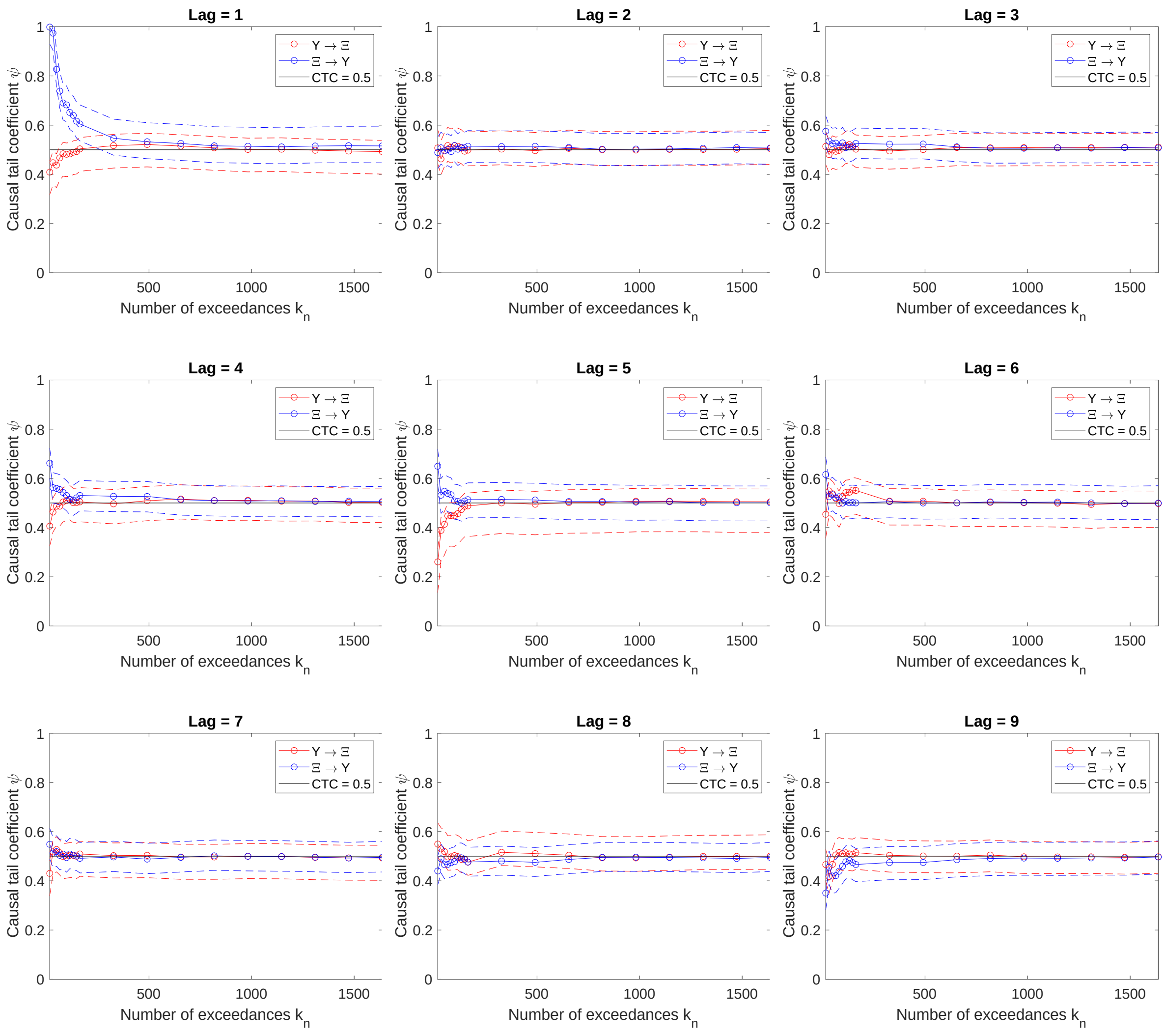

Figure S15: The estimated causal tail coefficient ψ with 95% bootstrap confidence intervals for variable Y and Ξ. The causal connection $\Xi \rightarrow Y$ detected for $Lag = 1$. The causal connection is affected by extrema, i.e., $\psi_{\Xi \rightarrow Y} = 1$.

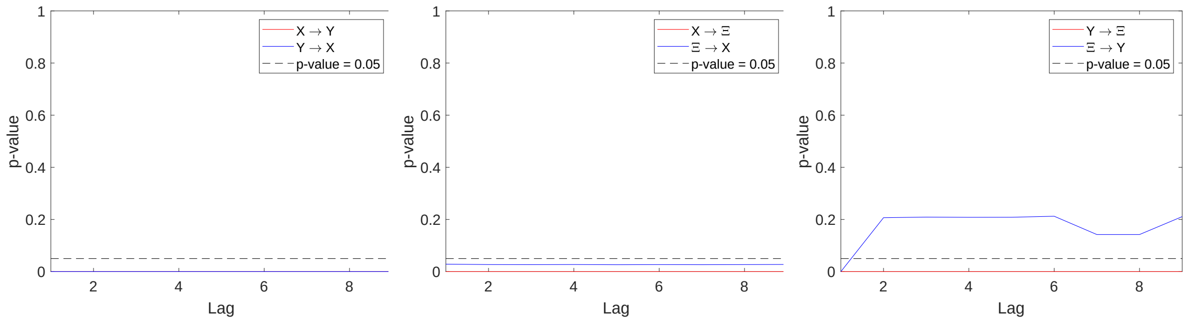

Figure S16: Results of Zanin's causality for the simulated time series X, Y, Ξ. The causal connections $X \leftrightarrow Y$, $X \leftrightarrow \Xi$, and $Y \rightarrow \Xi$ were detected for all considered lag values. The causal connection between $\Xi \rightarrow Y$ was detected only for $Lag = 1$.

	**GC**	**CTC**	**ZC**
X, Y	$X \leftrightarrow Y$	$X \rightarrow Y$	$X \leftrightarrow Y$
X, Ξ	$X \perp \Xi$	$X \rightarrow \Xi$	$X \leftrightarrow \Xi$
Y, Ξ	$\Xi \rightarrow Y$	$\Xi \rightarrow Y$	$Y \rightarrow \Xi$

Table S2: Results of causal analysis of time series X, Y, Ξ obtained by the Granger causality (GC), causal tail coefficients (CTC), and Zanin's causality (ZC).

Results of GC, CTC and ZC methods for the climate data

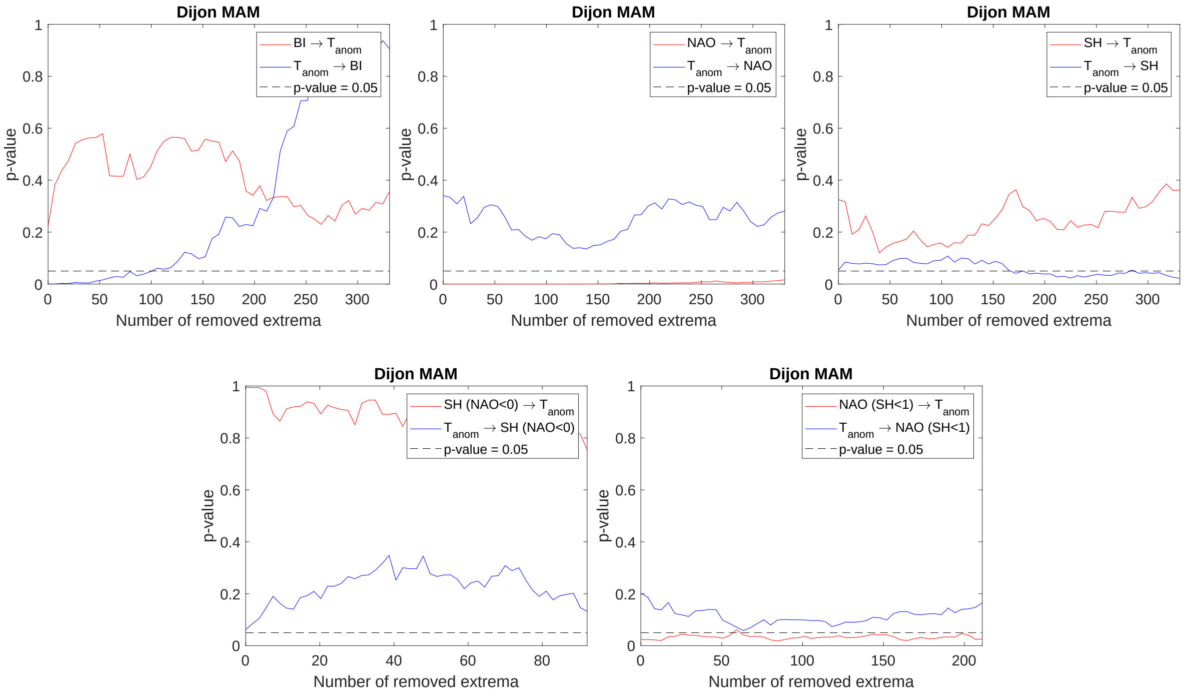

Figure S17: Results of the Granger causality between the temperature anomaly (T_{anom}) and the blocking index (BI), the North Atlantic Oscillation (NAO), the Siberian high index (SH), SH for negative NAO, NAO for SH lower than 1 in Dijon during MAM (March, April, May). The causal connection $NAO \rightarrow T_{anom}$, and $NAO(SH < 1) \rightarrow T_{anom}$ was detected independently of the number of removed extrema. After removing more than 1.5% of extrema values of BI, the connection $T_{anom} \rightarrow BI$ is not more present.

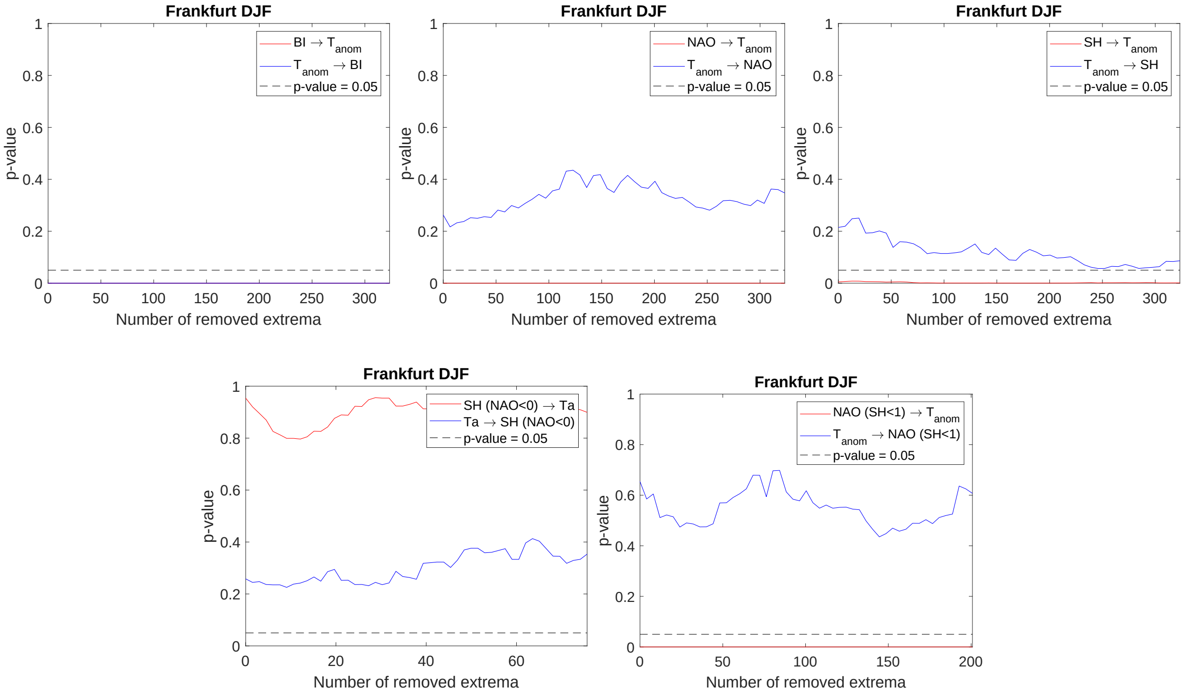

Figure S18: Results of the Granger causality between the temperature anomaly (T_{anom}) and the blocking index (BI), the North Atlantic Oscillation (NAO), the Siberian high index (SH), SH for negative NAO, NAO for SH lower than 1 in Frankfurt during DJF (December, January, February). The causal connection $BI \leftrightarrow T_{anom}$, $NAO \rightarrow T_{anom}$, $SH \rightarrow T_{anom}$, and $NAO(SH < 1) \rightarrow T_{anom}$ was detected independently of the number of removed extrema.

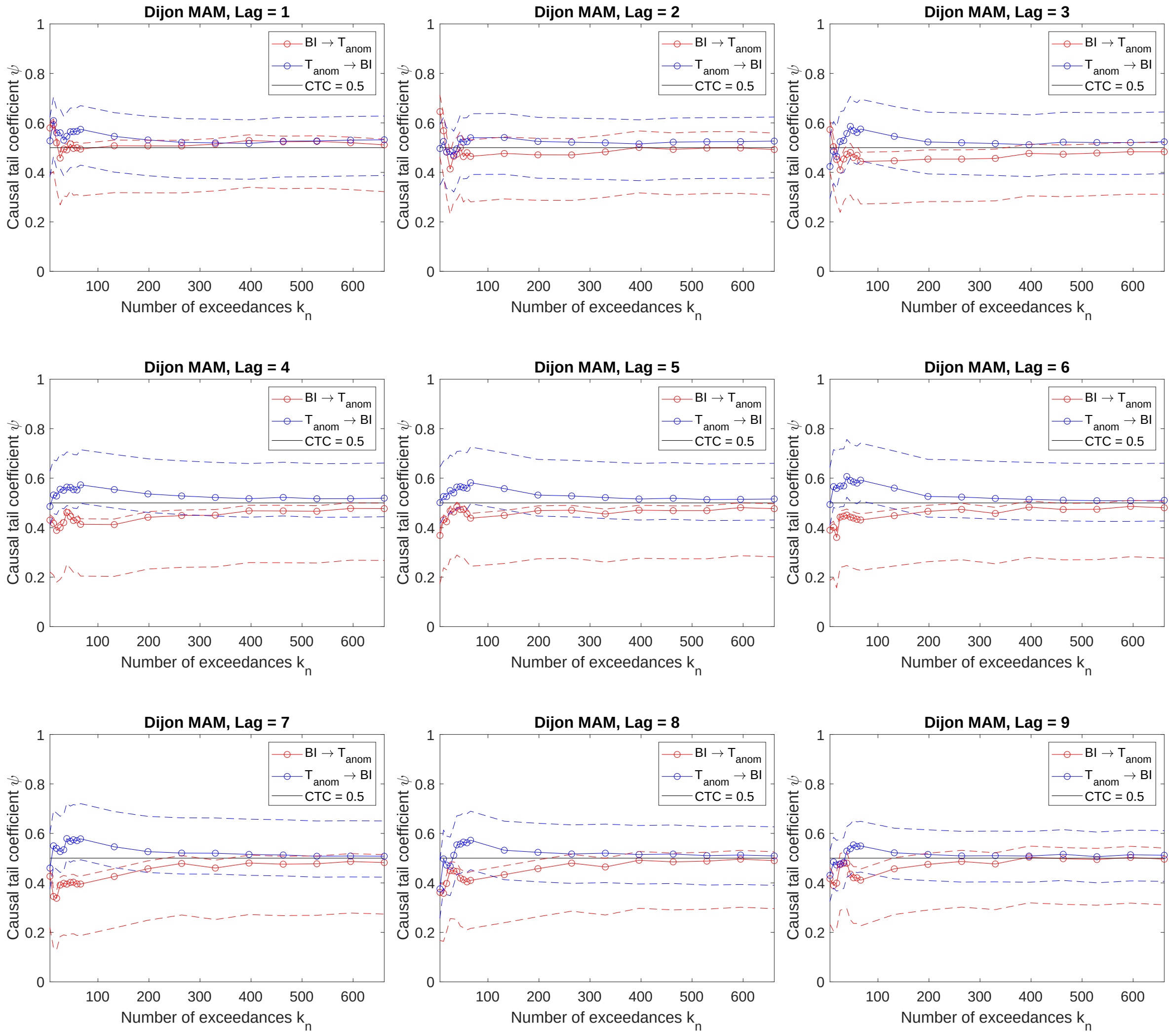

Figure S19: The estimated causal tail coefficient ψ with 95% bootstrap confidence intervals for blocking index (BI) and the temperature anomaly (T_{anom}) in Dijon during MAM for different values of exceedances k_n and considered $Lags$ of data. The absence of a causal connection between the variables BI and T_{anom} was concluded.

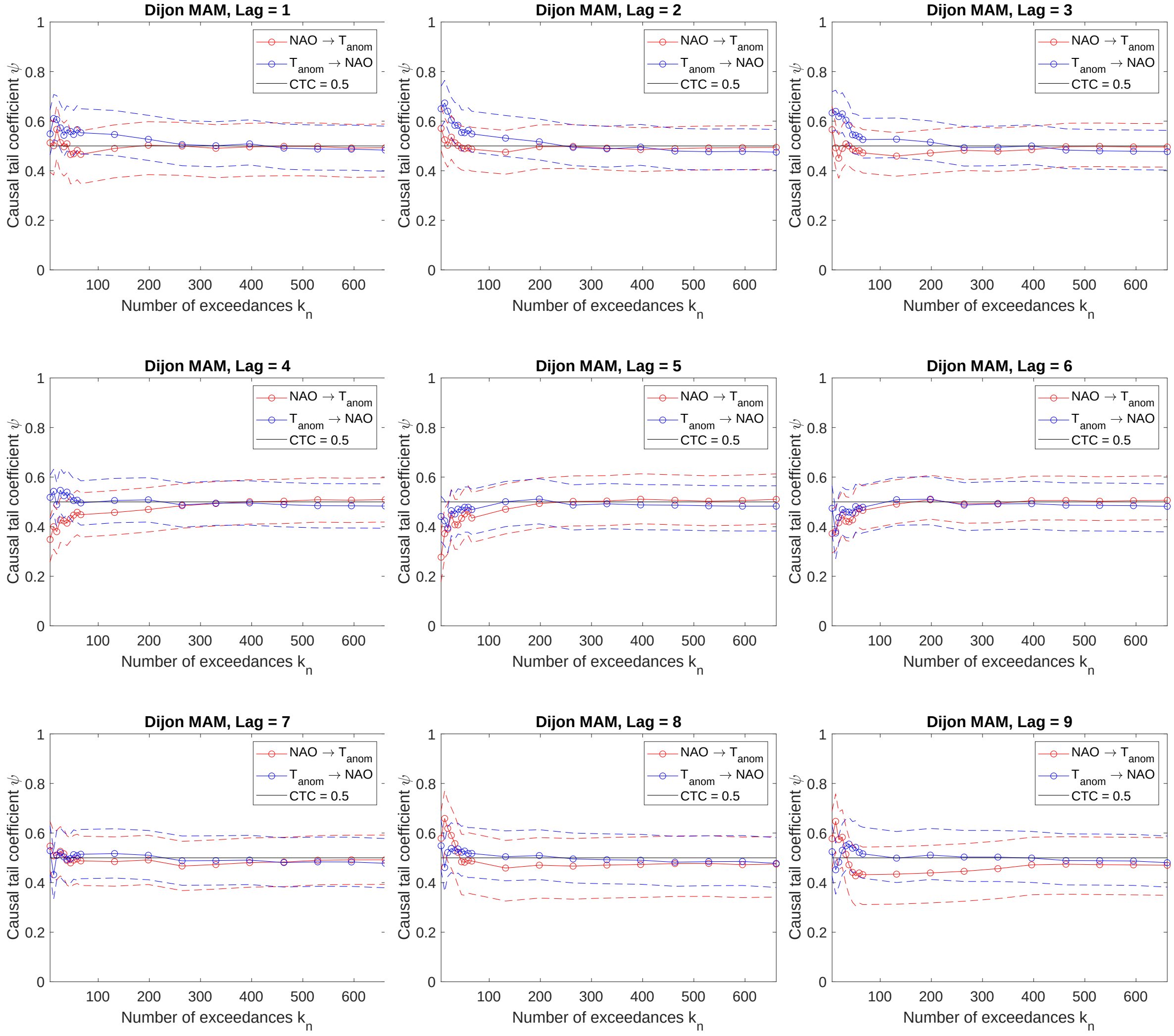

Figure S20: The estimated causal tail coefficient ψ between the North Atlantic Oscillation (NAO) and the temperature anomaly (T_{anom}) in Dijon during MAM for different values of exceedances k_n and considered Lag values. Since no ψ equals to 1, there is no causality between extremes, however $\psi_{T_{anom} \to NAO} > 0.5$ and $\psi_{NAO \to T_{anom}} = 0.5$ for small k_n values suggests causal connection $T_{anom} \to NAO$ for lags 2-3.

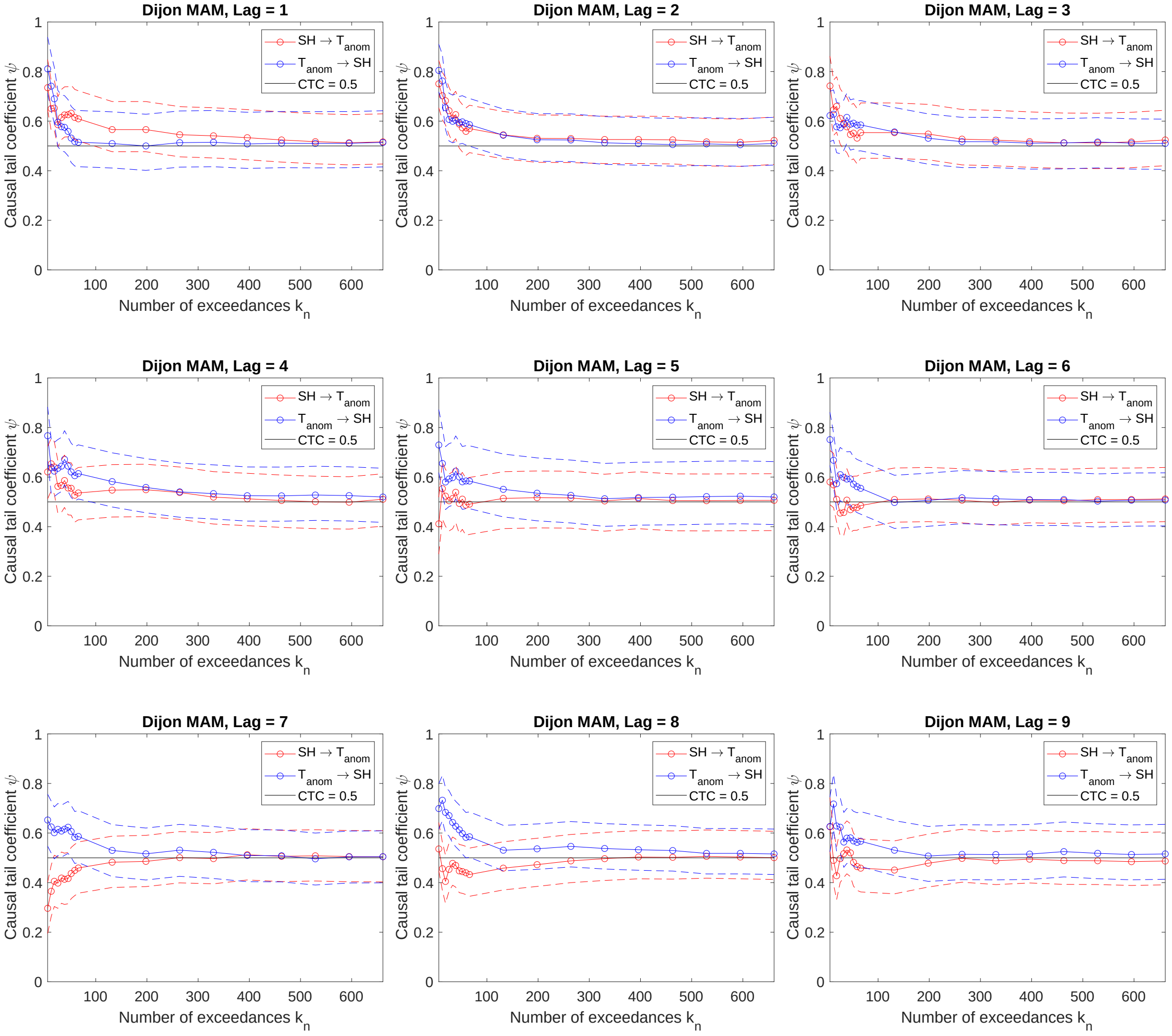

Figure S21: The estimated causal tail coefficient ψ with 95% bootstrap confidence intervals for the Siberian high index (SH) and the temperature anomaly (T_{anom}) in Dijon during MAM for different values of exceedances k_n and considered $Lags$ of data. Since no ψ equals to 1, there is no causality between extremes, however $\psi_{T_{anom} \to SH} > 0.5$, $\psi_{SH \to T_{anom}} > 0.5$ for small k_n values suggests bidirectional causal connection $T_{anom} \leftrightarrow SH$ for lags 1-4. On the other hand, $\psi_{T_{anom} \to SH} > 0.5$ and $\psi_{SH \to T_{anom}} = 0.5$ for small k_n values suggests causal connection $T_{anom} \to SH$ for lags 5-9.

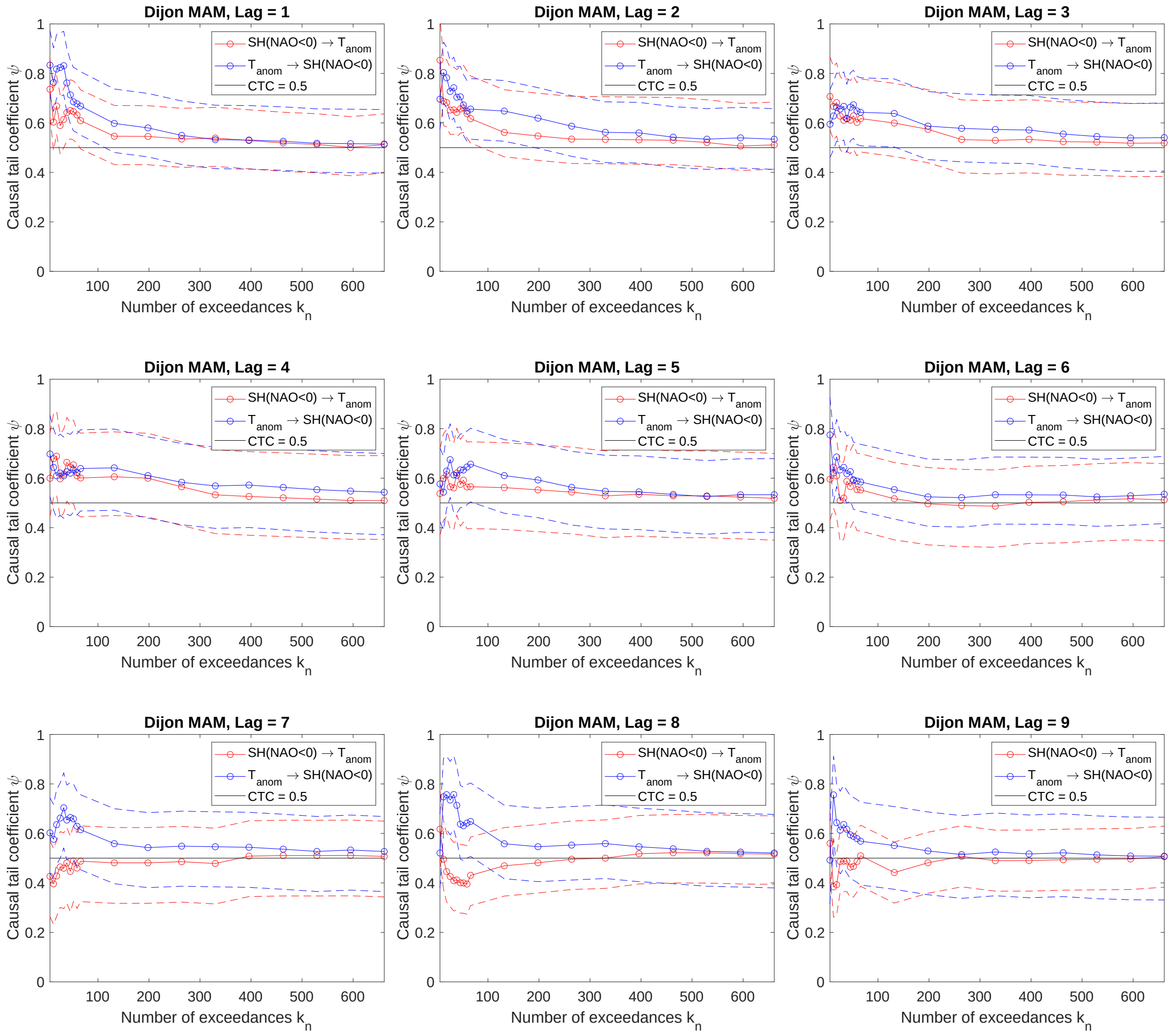

Figure S22: The estimated causal tail coefficient ψ between the Siberian high index (SH) for negative North Atlantic Oscillation (NAO) and the temperature anomaly (T_{anom}) in Dijon during MAM for different values of exceedances k_n and considered Lag values. Since no ψ equals to 1, there is no causality between extremes, however $\psi_{SH(NAO<0)\to T_{anom}} > 0.5$, $\psi_{T_{anom}\to SH(NAO<0)} > 0.5$ for small k_n values suggests bidirectional causal connection $T_{anom} \leftrightarrow SH$detected for lags $1-2$.

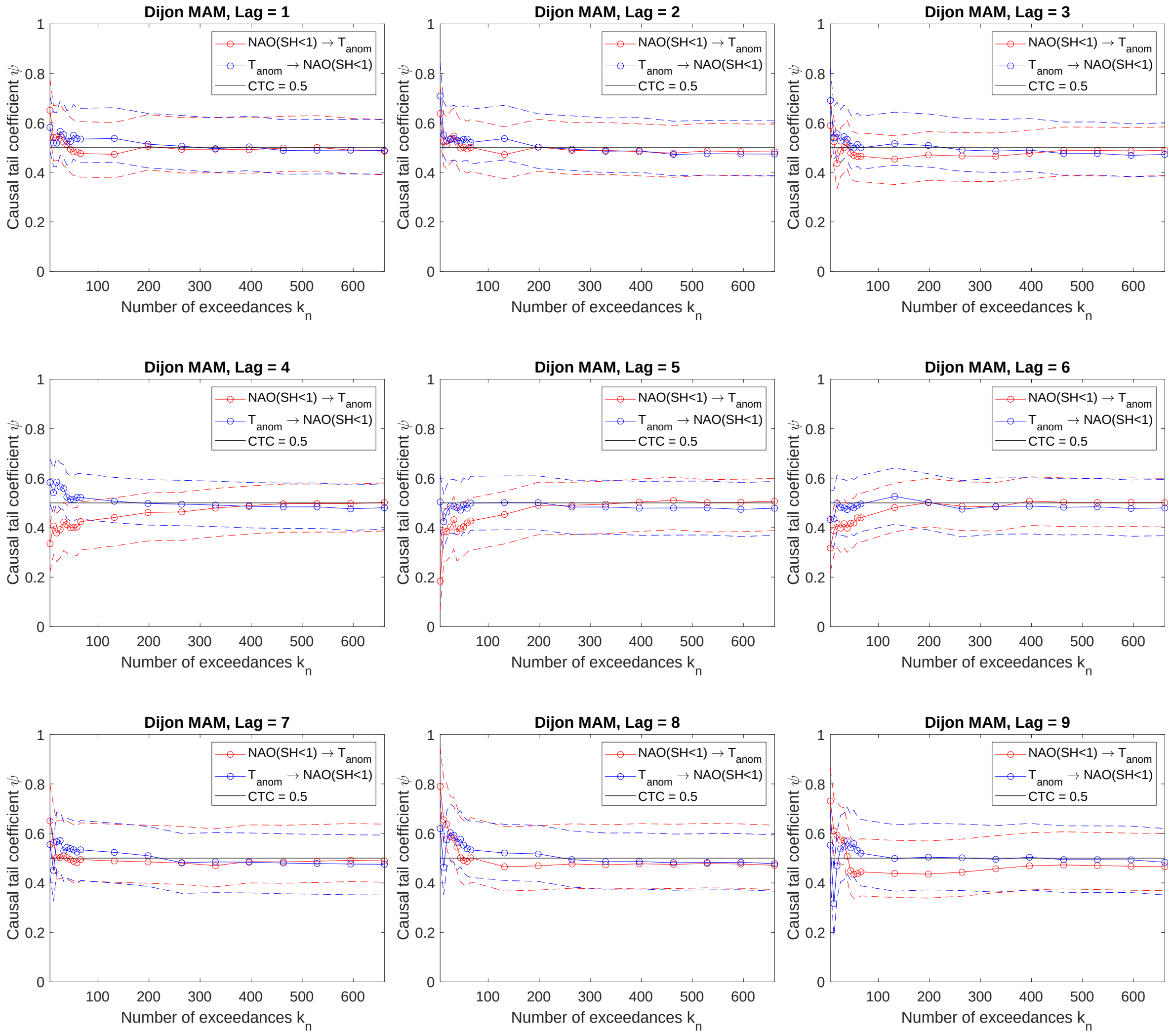

Figure S23: The estimated causal tail coefficient ψ with 95% bootstrap confidence intervals for the North Atlantic Oscillation (NAO) for the Siberian high index (SH) lower than 1 and the temperature anomaly (T_{anom}) in Dijon during MAM for different values of exceedances k_n and considered $Lags$ of data. The absence of causal connection between the variables $NAO(SH < 1)$ and T_{anom} was concluded.

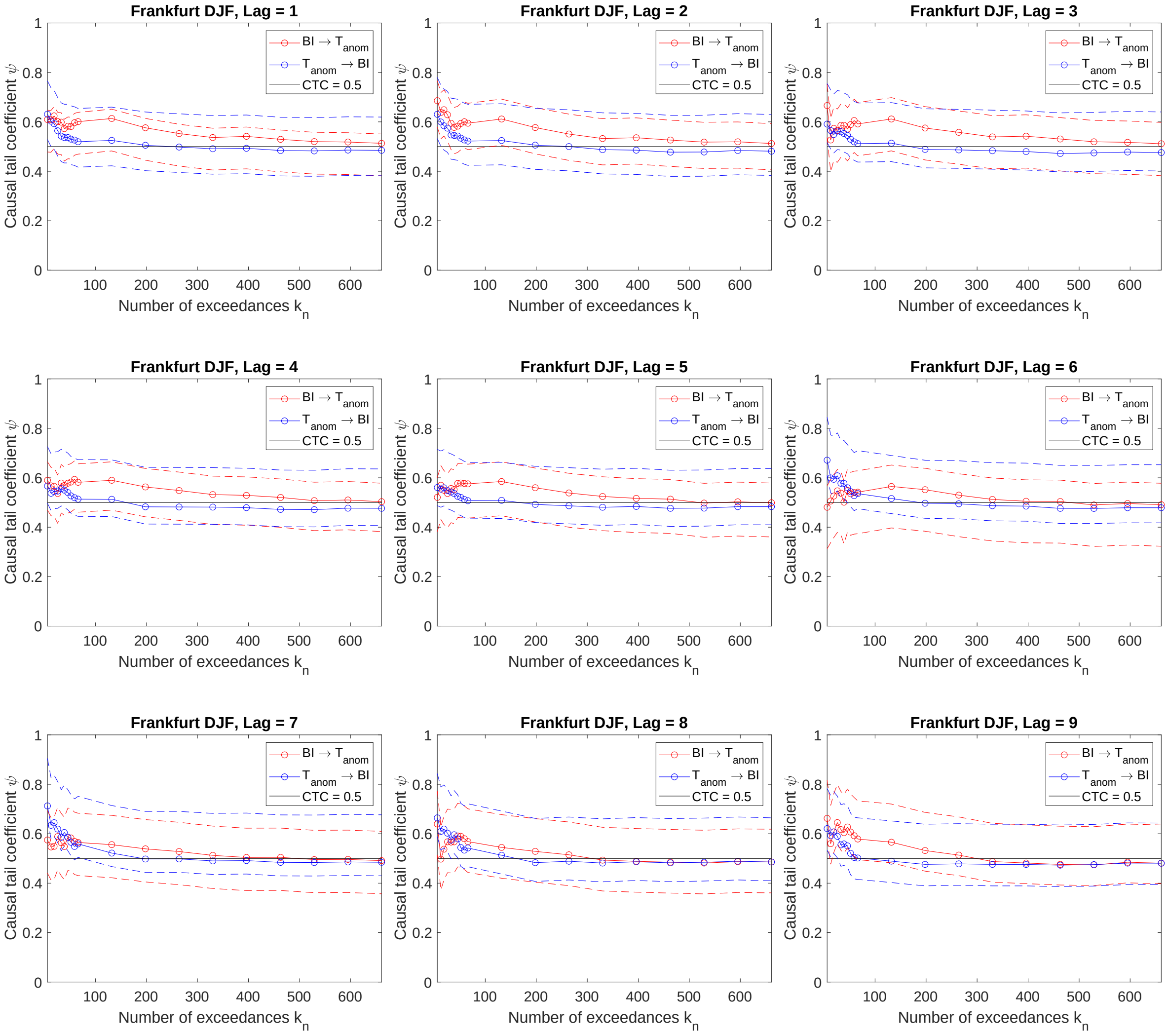

Figure S24: The estimated causal tail coefficient ψ with 95% bootstrap confidence intervals for the blocking index (BI) and the temperature anomaly (T_{anom}) in Frankfurt during DJF for different values of exceedances k_n and considered Lag values. Since no ψ equals to 1, there is no causality between extremes. However, $\psi_{BI \to T_{anom}} > 0.5$ and $\psi_{T_{anom} \to BI} > 0.5$ for small k_n values suggests bidirectional causal connection $T_{anom} \leftrightarrow BI$ for lag 2. On the other hand, $\psi_{T_{anom} \to BI} > 0.5$ and $\psi_{BI \to T_{anom}} = 0.5$ suggests causal connection $T_{anom} \to BI$ for $Lag = \{1, 6, 7, 8\}$ and small k_n values.

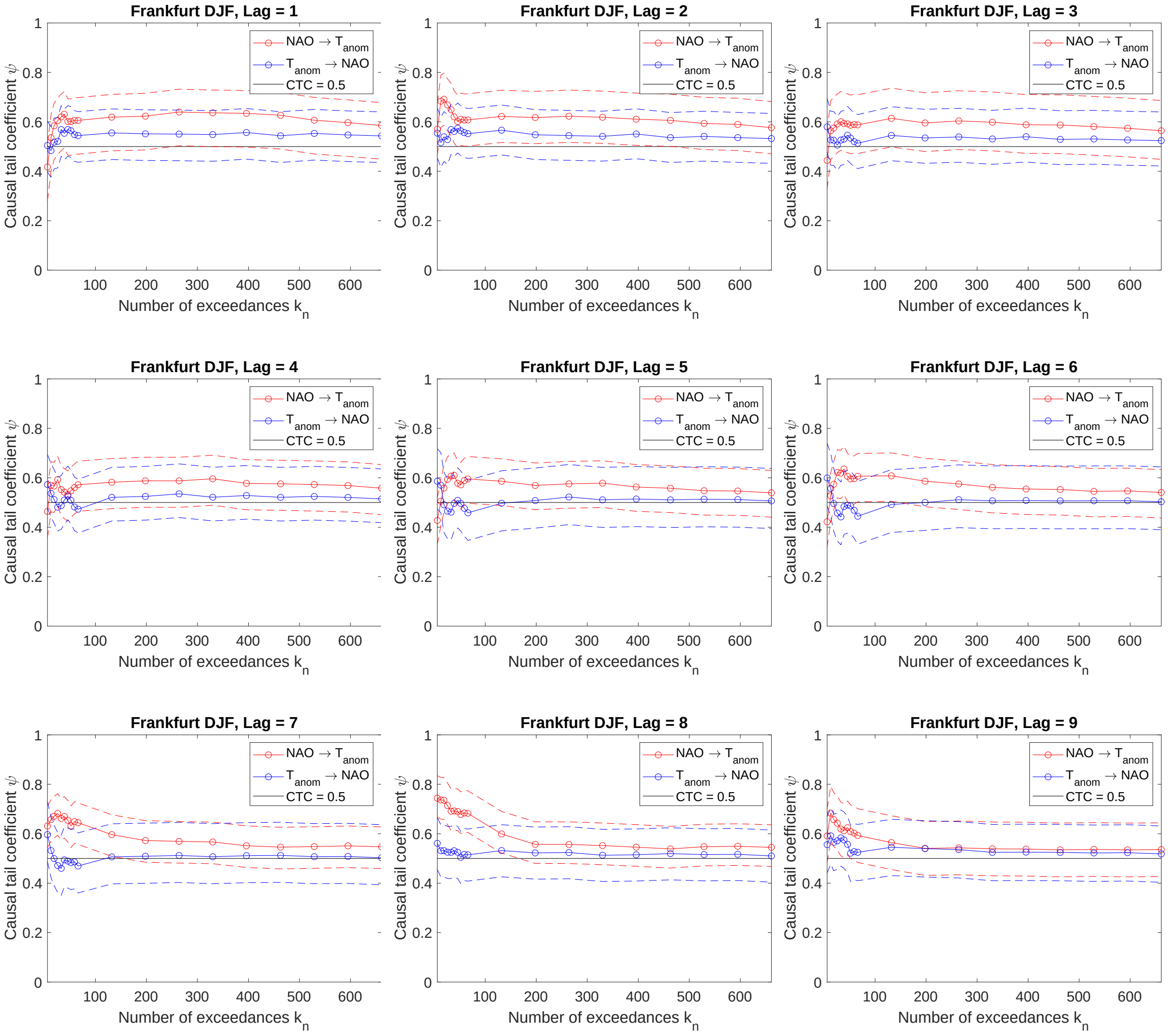

Figure S25: The estimated causal tail coefficient ψ with 95% bootstrap confidence intervals for the North Atlantic Oscillation (NAO) and the temperature anomaly (T_{anom}) in Frankfurt during DJF for different values of exceedances k_n and considered Lag values. Since no ψ equals to 1, there is no causality between extremes. However, $\psi_{NAO \to T_{anom}} > 0.5$ and $\psi_{T_{anom} \to NAO} = 0.5$ for small k_n values suggests causal connection $NAO \to T_{anom}$ for lag $7-8$.

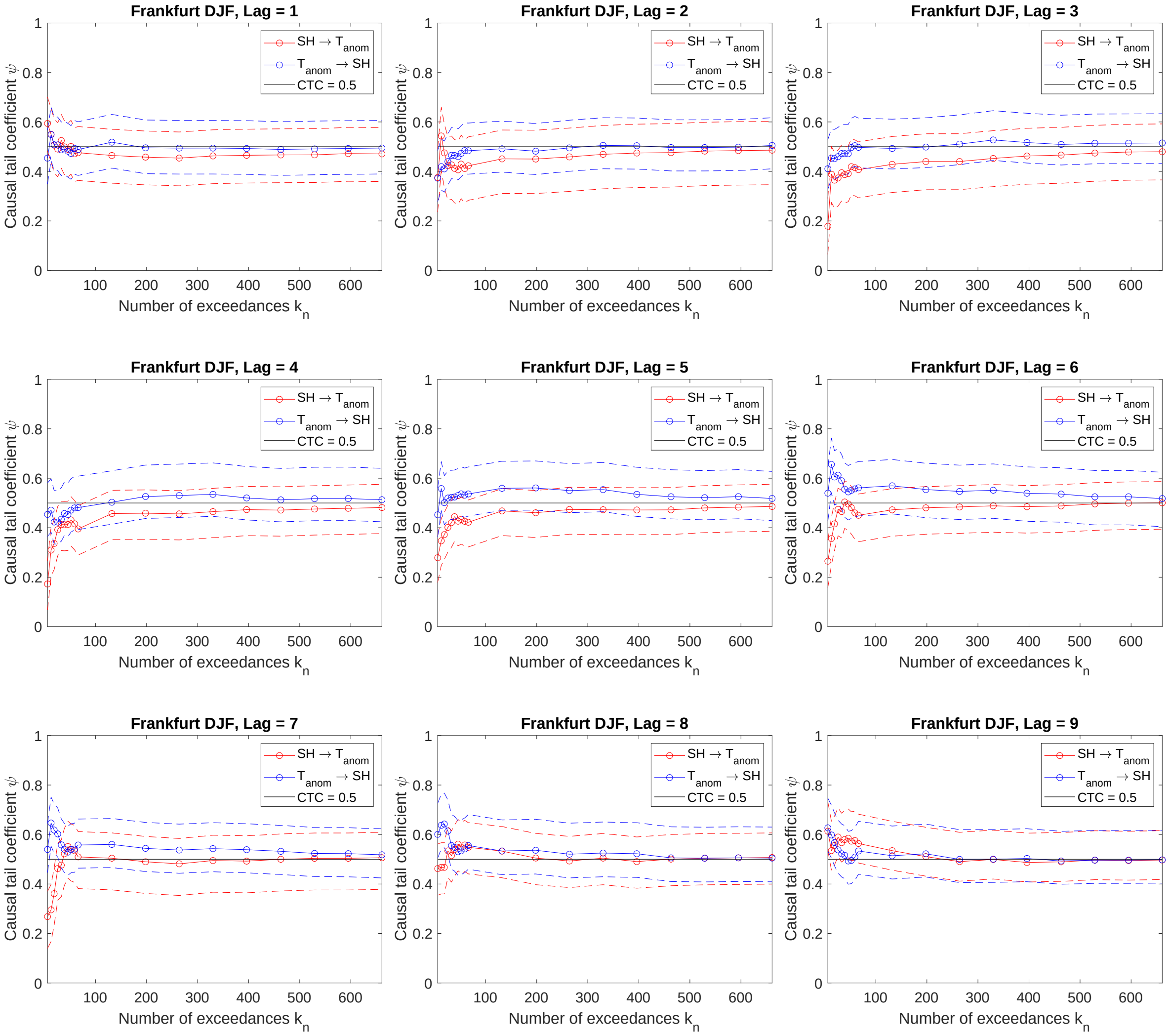

Figure S26: The estimated causal tail coefficient ψ with 95% bootstrap confidence intervals for the Siberian high index (SH) and the temperature anomaly (T_{anom}) in Frankfurt during DJF for different values of exceedances k_n and considered Lag values. The absence of a causal connection between the variables SH and T_{anom} was concluded.

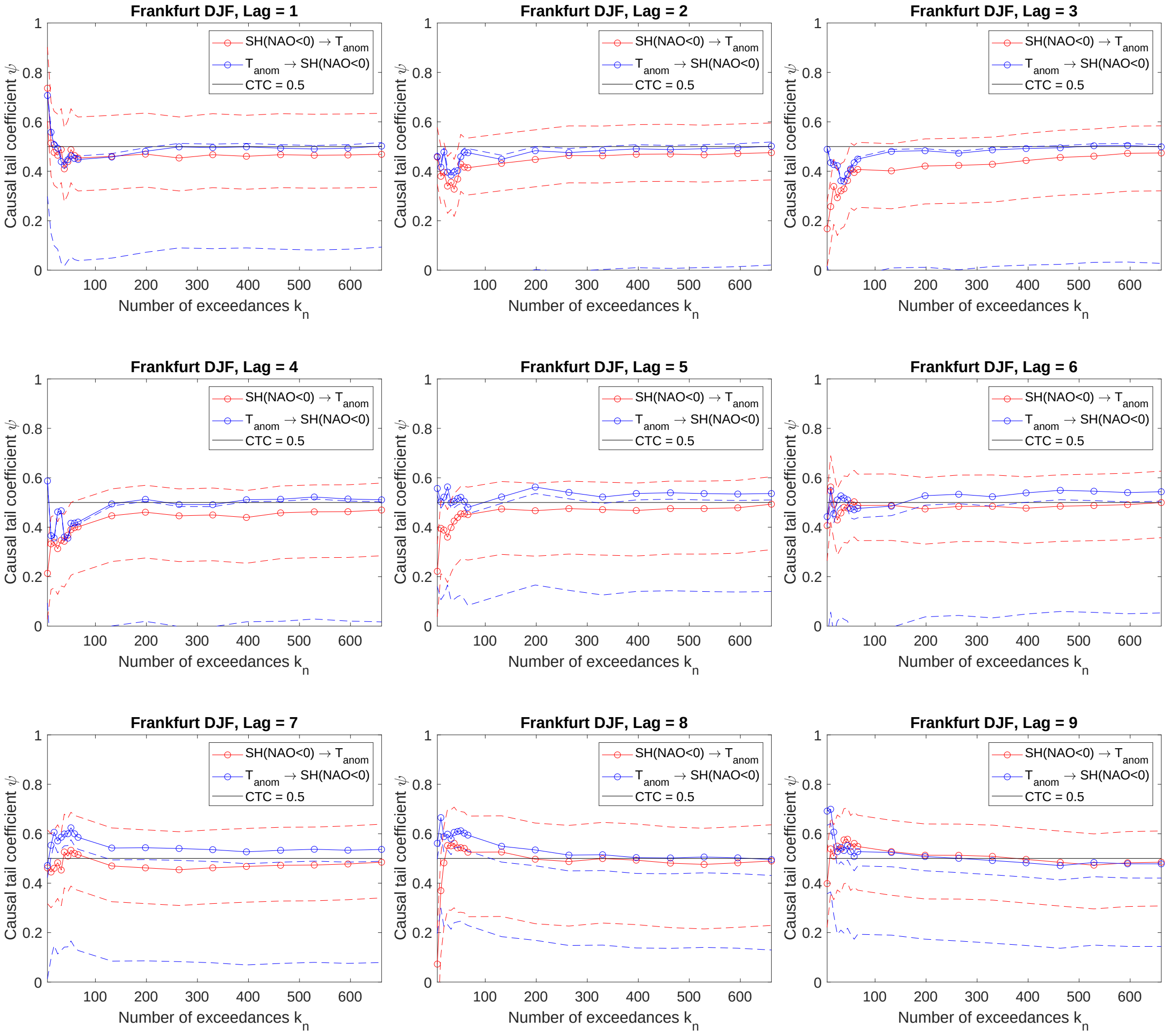

Figure S27: The estimated causal tail coefficient ψ with 95% bootstrap confidence intervals for the Siberian high index (SH) for negative North Atlantic Oscillation (NAO) and the temperature anomaly (T_{anom}) in Frankfurt during DJF for different values of exceedances k_n and considered Lag values. The absence of a causal connection between the variables $SH(NAO < 0)$ and T_{anom} was concluded.

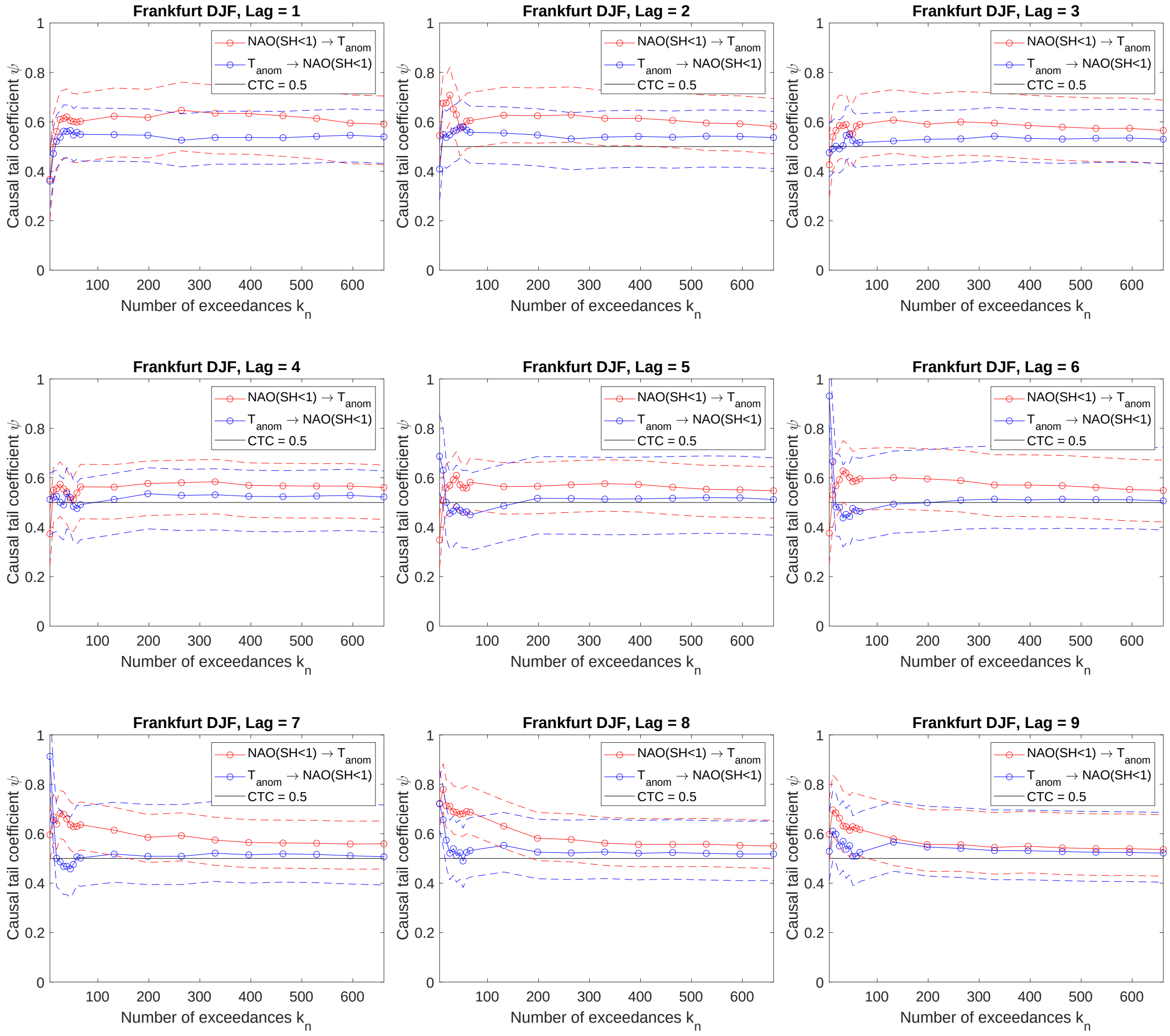

Figure S28: The estimated causal tail coefficient ψ with 95% bootstrap confidence intervals for the North Atlantic Oscillation (NAO) for the Siberian high index (SH) lower than 1 and the temperature anomaly (T_{anom}) in Frankfurt during DJF for different values of exceedances k_n and considered Lag values. Since no ψ equals to 1, there is no causality between extremes. However, $\psi_{T_{anom} \to NAO(SH<1)} > 0.5$ and $\psi_{NAO(SH<1) \to T_{anom}} = 0.5$ for small k_n values suggests causal connection $T_{anom} \to NAO(SH < 1)$ for lag $5-7$. On the other hand, $\psi_{T_{anom} \to NAO(SH<1)} > 0.5$ and $\psi_{NAO(SH<1) \to T_{anom}} > 0.5$ for small k_n values suggests bidirectional causal connection $NAO(SH < 1) \leftrightarrow T_{anom}$ for lag 8.

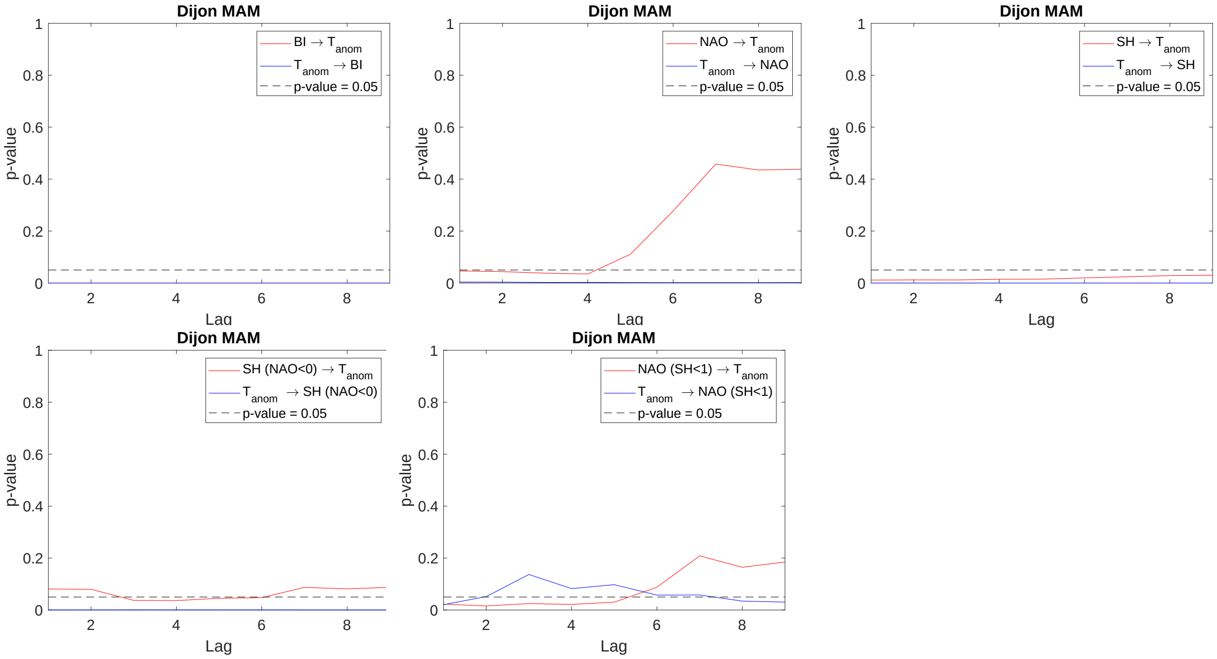

Figure S29: Results of Zanin's causality between the temperature anomaly (T_{anom}) and the blocking index (BI), the North Atlantic Oscillation (NAO), the Siberian high index (SH), SH for negative NAO, NAO for SH lower than 1 in Dijon during MAM for different Lag values. The bidirectional causal connection $BI \leftrightarrow T_{anom}$ and $SH \leftrightarrow T_{anom}$ was detected independently of a lag value. The causal connection between T_{anom} and NAO, $SH(NAO < 0)$, $NAO(SH < 1)$ depends on a lag value.

	T_{Dijon}			$T_{Frankfurt}$		
	GC	CTC	ZC	GC	CTC	ZC
BI	$\leftarrow$	$\perp$	$\leftrightarrow$	$\leftrightarrow$	$\leftarrow^*$	$\leftrightarrow$
NAO	$\rightarrow$	$\leftarrow$	$\leftrightarrow^*$	$\rightarrow$	$\rightarrow$	$\leftrightarrow$
SH	$\perp$	$\leftrightarrow^*$	$\leftrightarrow$	$\rightarrow$	$\perp$	$\rightarrow$
$SH(NAO < 0)$	$\perp$	$\leftrightarrow$	$\leftarrow^*$	$\perp$	$\perp$	$\leftrightarrow$
$NAO(SH < 1)$	$\rightarrow$	$\perp$	$\rightarrow^*$	$\rightarrow$	$\leftarrow^*$	$\rightarrow$

Table S3: Summary of causal analysis results obtained by the Granger causality (GC), causal tail coefficients (CTC), and Zanin's causality (ZC) between the blocking index (BI), the North Atlantic Oscillation (NAO), the Siberian high index (SH), SH for negative NAO, NAO for SH lower than 1 and the temperature anomaly in Dijon during MAM (T_{Dijon}) and in Frankfurt during DJF ($T_{Frankfurt}$). (*) - detected causal connection depends on a lag value.

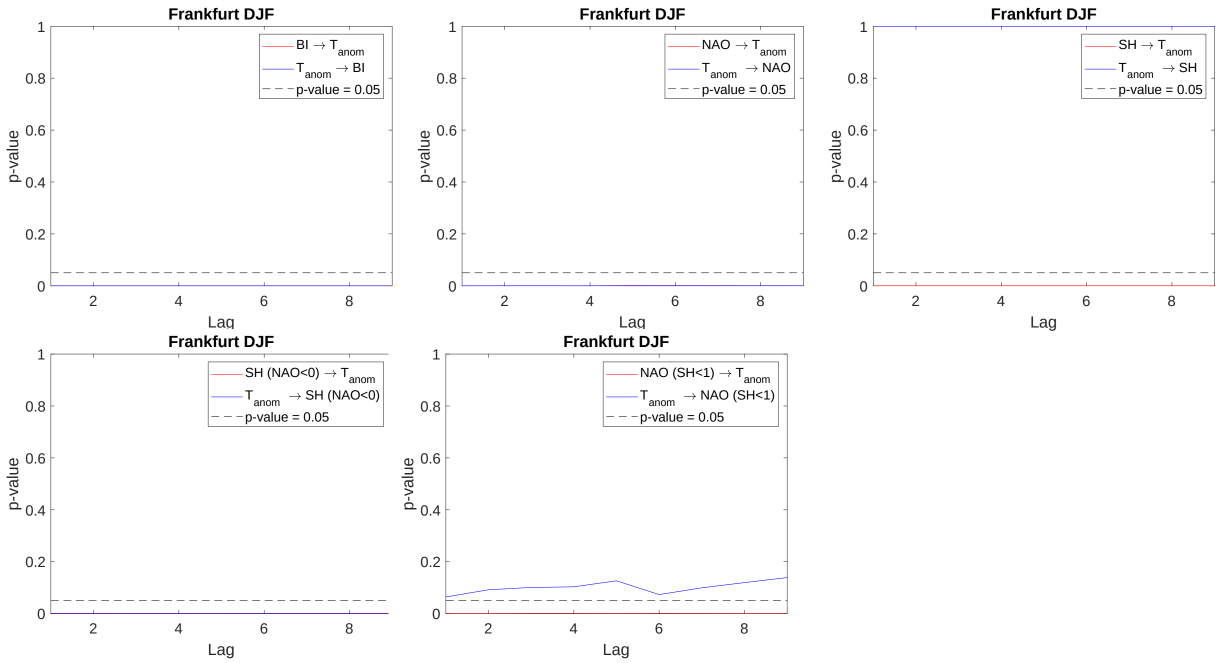

Figure S30: Results of Zanin's causality between the temperature anomaly (T_{anom}) and the blocking index (BI), the North Atlantic Oscillation (NAO), the Siberian high index (SH), SH for negative NAO, NAO for SH lower than 1 in Frankfurt during DJF for different Lag values. The bidirectional causal connection $BI \leftrightarrow T_{anom}$, $NAO \leftrightarrow T_{anom}$, $SH(NAO < 0) \leftrightarrow T_{anom}$ and unidirectional causal connection $SH \rightarrow T_{anom}$, $NAO(SH < 1) \rightarrow T_{anom}$ were detected independently of a lag value.
